# LC–HRMS Lipidomic Fingerprints in Serbian Cohort of Schizophrenia Patients

**DOI:** 10.3390/ijms251910266

**Published:** 2024-09-24

**Authors:** Suzana Marković, Milka Jadranin, Zoran Miladinović, Aleksandra Gavrilović, Nataša Avramović, Marija Takić, Ljubica Tasic, Vele Tešević, Boris Mandić

**Affiliations:** 1 University of Belgrade—Faculty of Chemistry, Studentski trg 12–16, 11000 Belgrade, Serbia; suzana.markovic@med.bg.ac.rs (S.M.); vtesevic@chem.bg.ac.rs (V.T.); 2 University of Belgrade—Faculty of Medicine, Institute of Forensic Medicine, Deligradska 31a, 11000 Belgrade, Serbia; 3 University of Belgrade—Institute of Chemistry, Technology and Metallurgy, Department of Chemistry, Njegoševa 12, 11000 Belgrade, Serbia; milka.jadranin@ihtm.bg.ac.rs; 4 Institute of General and Physical Chemistry, Studentski trg 12–16, 11158 Belgrade, Serbia; zmiladinovic@iofh.bg.ac.rs; 5 Special Hospital for Psychiatric Diseases “Kovin”, Cara Lazara 253, 26220 Kovin, Serbia; gavrilovicaleksandra74@gmail.com; 6 University of Belgrade—Faculty of Medicine, Institute of Medical Chemistry, Višegradska 26, 11000 Belgrade, Serbia; natasa.avramovic@med.bg.ac.rs; 7 University of Belgrade—Institute for Medical Research, National Institute of Republic of Serbia, Center of Research Excellence for Nutrition and Metabolism, Group for Nutrition and Metabolism, Tadeuša Košćuška 1, 11000 Belgrade, Serbia; marija.takic@imi.bg.ac.rs; 8 Institute of Chemistry, Organic Chemistry Department, Universidade Estadual de Campinas, UNICAMP, Campinas 13083-970, SP, Brazil; ljubica@unicamp.br

**Keywords:** biomarkers, schizophrenia, lipidomics, mass spectrometry, serum

## Abstract

Schizophrenia (SCH) is a major mental illness that causes impaired cognitive function and long-term disability, so the requirements for reliable biomarkers for early diagnosis and therapy of SCH are essential. The objective of this work was an untargeted lipidomic study of serum samples from a Serbian cohort including 30 schizophrenia (SCH) patients and 31 non-psychiatric control (C) individuals by applying liquid chromatography (LC) coupled with high-resolution mass spectrometry (HRMS) and chemometric analyses. Principal component analysis (PCA) of all samples indicated no clear separation between SCH and C groups but indicated clear gender separation in the C group. Multivariate statistical analyses (PCA and orthogonal partial least squares discriminant analysis (OPLS-DA)) of gender-differentiated SCH and C groups established forty-nine differential lipids in the differentiation of male SCH (SCH-M) patients and male controls (C-M), while sixty putative biomarkers were identified in the differentiation of female SCH patients (SCH-F) and female controls (C-F). Lipidomic study of gender-differentiated groups, between SCH-M and C-M and between SCH-F and C-F groups, confirmed that lipids metabolism was altered and the content of the majority of the most affected lipid classes, glycerophospholipids (GP), sphingolipids (SP), glycerolipids (GL) and fatty acids (FA), was decreased compared to controls. From differential lipid metabolites with higher content in both SCH-M and SCH-F patients groups compared to their non-psychiatric controls, there were four common lipid molecules: ceramides Cer 34:2, and Cer 34:1, lysophosphatidylcholine LPC 16:0 and triacylglycerol TG 48:2. Significant alteration of lipids metabolism confirmed the importance of metabolic pathways in the pathogenesis of schizophrenia.

## 1. Introduction

Schizophrenia (SCH) is a complex psychiatric disease with characteristic symptoms, including positive symptoms, negative symptoms, and impairments in cognition, that result in long-term disability and invalidity [1,2,3]. Lifetime prevalence is about 1%, which amounts to about 24 million people worldwide [4]. First symptoms most often occur in adolescence or early adulthood and it is very important to provide early diagnosis and appropriate treatment [5]. Specifically, during these formative years of SCH, the clinical characteristics may be general and shared with other mental illnesses, such as anxiety, obsessive-compulsive disorder, depression and bipolar disorder, so that, due to the heterogeneity of symptoms, distinguishing between diagnoses is a huge challenge [6,7]. The diagnosis of SCH is based on subjective clinical assessments of complex symptoms considering patient reports, reports of their families, and on specific diagnostic scales. So far, there is no clinical test of biomarkers identification for any mental disease [8,9]. Therefore, exploration and identification of biomarkers have a pivotal role in early and accurate diagnosis, as well as monitoring of clinical SCH treatment [9].

After adipose tissue, lipids are the second most abundant biomolecules in the brain [10]. Lipids have a very important structural and functional role in many essential processes, whose alteration can cause damage to the central nervous system (CNS), development of psychiatric disorders, and their pathogenesis [11]. Lipids are the main structural components of cell membranes, and they are involved in many processes in the brain cells, such as myelination, neurotransmission, synaptic plasticity, energy metabolism and inflammatory processes [10]. Alterations in the glycerophospholipid (GP), sphingolipid (SP), and glycerolipid (GL) metabolic pathways in the plasma and serum of SCH patients have been depicted [12,13,14,15,16,17,18,19], as well as in the arachidonic acid (AA) metabolism in the brain and periphery [20,21,22].

In recent years, ‘omics’ technologies, such as genomics, proteomics and metabolomics, have been of crucial importance for biomarkers determination in mental diseases, and combined data from these platforms represent a future perspective for a comprehensive view of SCH diagnosis [23,24,25,26,27,28,29,30,31,32]. Amongst all these platforms, lipidomics is still not sufficiently enough explored [33,34,35]. Lipidomics, based on the application of liquid chromatography combined with high resolution mass spectroscopy (LC–HRMS), is a powerful tool for identification and quantification of important lipid molecules in blood (plasma and serum) that can provide comprehensive insight into SCH pathology [36,37,38,39,40,41].

The main goal of this study was to accomplish untargeted lipidomics of serum samples from gender-differentiated groups, SCH patients (males, SCH-M and females, SCH-F) and non-psychiatric control individuals (males, C-M and females, C-F), to explore and compare their lipid profiles, as well as to analyze differential lipids between SCH-M and C-M and between SCH-F and C-F groups. These results may shed light on alterations in lipid metabolism in SCH with the aim of identifying potential biomarkers, as well as improving future research on the diagnosis and treatment of this disease.

## 2. Results

A total of 192 *m*/*z* features for combined negative and positive ion modes, and 169 chromatograms (after removal of 14 distinctive outliers), obtained by using the LC–HRMS method, were included in the final dataset. The multivariate statistical analyses (principal component analysis (PCA) and orthogonal partial least squares discriminant analysis (OPLS-DA)) were used for these SCH lipidomics studies.

### 2.1. PCA Analysis

Apart from robust PCA models feasible for outliers’ identification [42,43,44], PCA analysis considering the overall variability among observations in the dataset could also be performed. To extract the important information from the data table and to express this information as a set of new orthogonal variables called principal components, analysis of so-called unsupervised models was performed, resulting in pattern similarity (or dissimilarity) of the observation and the variables [45], at the same time.

**Figure 1:**
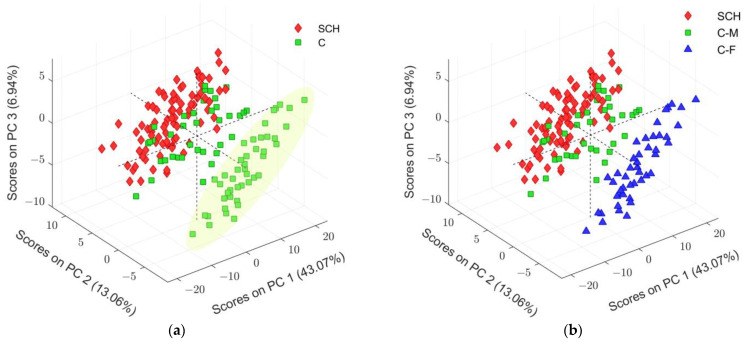
(**a**) PCA model of log10 transformed and auto-scaled dataset, represented by first three PC components. Main classes, schizophrenia (SCH) and non-psychiatric control (C) groups of observations are assigned with red diamonds and green squares, respectively. Pale green ellipse encompasses observation in C with distinctive clustering within this class; (**b**) the same PCA model where, in comparison to the model presented in (**a**), the non-psychiatric control class was additionally sub-assigned by different colors according to gender identity (green squares for males: C-M and blue triangles for females: C-F).

After excluding the outliers, the six components PCA model was accomplished with log10 transformed and auto-scaled (without normalization) data. The number of relevant components in each of the PCA models was determined based on the Scree plot and the minimum of the Root Mean Squared Error of Cross-Validation (RMSECV) (Figure 1).

**Figure 2:**
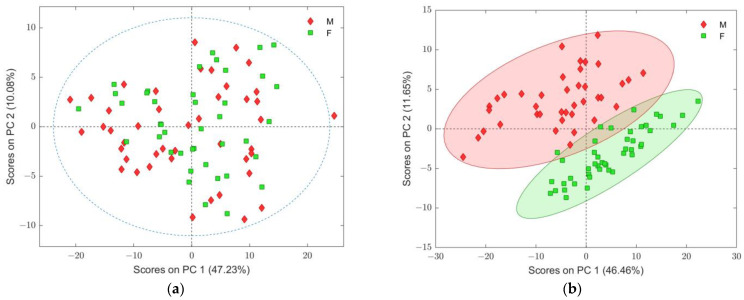
(**a**) Score plot of PCA model of schizophrenia (SCH) main class only, taking into consideration the first two PC components; (**b**) score plot of PCA model of non-psychiatric control (C) main class only. Confidence ellipse level rounding each sub-class is 95%. In both cases, sub-classes were assigned to gender identity (males: M with red diamonds and females: F with green squares).

The clearly identified group of observations in the non-psychiatric control class forms a distinctive pattern (the cluster is rounded with a pale green ellipse in Figure 1a) in comparison with other observations from the same class. Such a peculiar sample arrangement of scores only inside one class merits particular attention. Subsequent reassigning of observation according to gender relevance in class C, as depicted in Figure 1b, enables better insight into the potential reasons responsible for the observed arrangement in the PCA score plot. Therefore, new PCA models for each main class separately were assembled based on the gender categorical variable for sub-class assignment in both cases (Figure 2). Models were composed using all predictors indicated in the initial data table, as well as with excluded observations previously identified as outliers.

PCA scores plot of SCH class (Figure 2a), where gender classes were indicated, did not show any visible distinctive pattern in any of the specified sub-classes. Furthermore, all scores of observations inside the SCH class were distributed around the center of the PCA space (Figure 2a). On the other hand, the PCA scores plot for C class, depicted in Figure 2b, clearly showed distinctive separation between genders. Moreover, the female (F) samples were tightly grouped. Therefore, the overlapped samples of C-M with the SCH class (Figure 1b) could be a consequence of lipid hallmarks among C-F samples.

The obtained result undoubtedly suggested that a number of features contribute more significantly to the separation between male and female members of the class of non-psychiatric control observation exclusively, but without any similar effect recognizable in the gender members in the schizophrenia group.

Therefore, to provide a better understanding of the underlying process that generated the data for the C group of samples, the entire C class was extracted from the initial data table and treated independently in the sense of a supervised classification algorithm. Representative classes were assigned according to gender identity (categorical variable) inside the main C class (i.e., male—M and female—F), and the resulting data table comprised 84 observations of 31 unique individuals after outlier exclusion pre-treatment. The primary goal was to achieve identification of variables according to their relevance in position or position ranking, rather than determine a subset of features which would improve prediction performance of the classifier.

The simplest variable ranking filter metrics represented the Fisher or F-ratio, which explained the pooled variance of all samples over the variance attributable to class means, relative to overall mean [46]. The F-ratio is described by the F-distribution, which included degree-of-freedom values for both the numerator and denominator as (g − 1) and (n − g), respectively, as input parameters, where n is the total number of samples and g refers to the number of classes. Critical values of significance were used to evaluate the appropriate threshold based on the parametric F-Distribution and are shown in Table S1 (Supplementary Material) [46]. From the obtained values, a threshold of 149 relevant features was determined based on probability *p* < 0.05 and enabling the separation between two sub-classes of PCA scores plot (Figure 2b).

### 2.2. Variable Importance from Random Forest Classifier

**Figure 3:**
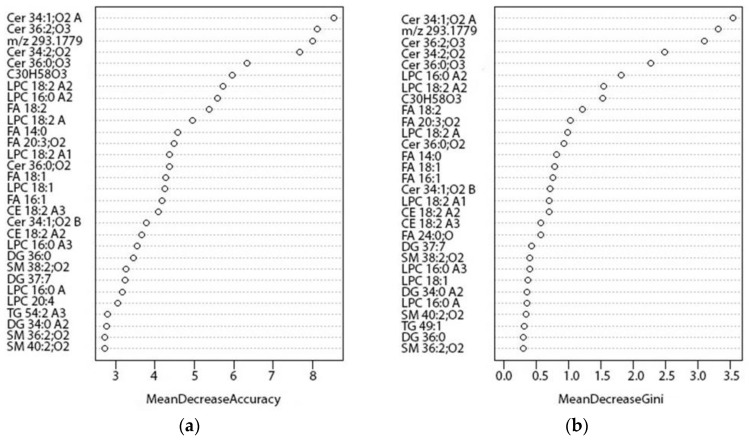
Variable importance of the first 30 features ranked according to (**a**) Mean Decrease Accuracy and (**b**) Mean Decrease Gini obtained from Random Forest classification model. Cer: *N*-acylsphinganines (dihydroceramides); LPC: 1-acyl-sn-glycero-3-phosphocholines; FA: fatty acids; CE: cholesterol esters; SM: ceramide phosphocholines (sphingomyelins); DG: diacylglycerols; TG: triacylglycerols.

The problem of stability (measure of robustness to data perturbation) of the ranked feature list resulted in the obtaining of drastically different biomarkers with the slightest perturbation of the dataset [47]. To assess the stability of variable ranking and perform a robust variable ranking, resampling techniques (with or without replacement) were applied to the original training set to produce N different versions of the training sets [47,48]. Then, some variable ranking algorithms were applied to evaluate the order of features, i.e., ranking position. The Random Forest classifier [49,50] almost entirely satisfied the resampling precondition, providing at the same time a reliable variable importance list based on two different metrics (measures of significance), Mean Decrease Gini and Mean Decrease Accuracy. Each tree was constructed using a different bootstrap sample from the original data, where about one-third of the observations were left out of the bootstrap sample and not used in the construction of the tree, known as the out-of-bag [50], or disjoint test set, for each tree. The mean decrease in Gini coefficient represented a measure of how each variable contributes to the homogeneity of the nodes and leaves in the resulting Random Forest. This measure, also known as Mean Decrease Gini or Gini importance [51], represented how often a variable was used to split the data across multiple decision trees, where Mean Decrease Accuracy described the difference in prediction accuracy when a considered variable is excluded from the model [46]. Either of these is used to evaluate variable importance and the subsequent ranking list for each Random Forest classifier [51]. The results of variable importance ranking for the first 30 features obtained from the Random Forest classifier [52] are presented in Figure 3. Variable importance accordant to *p*-values with corrected Gini importance was computed for 1000 generated classifiers, based on Janitza’s method from the R package “ranger” [53]. Since the obtained values of significance (*p* < 0.05) followed normal distribution, threshold was determined with confidence interval in 93–135 (114 ± 21) of the ranked features.

However, observations which are under consideration included replicated samples (triplicates) for each of the individuals and therefore there was a possibility that during the process of random resampling (through the Random Forest algorithm), some individuals contributed more frequently with belonging replicates than others from the same data table. This undoubtedly led to biased sampling and consequently to some discrepancy expected between successive classifying variable ranking lists. A straightforward solution, which we proposed in this work, was that, before each Random Forest classification, random sub-sampling was applied, where each of the selected samples was randomly selected among all possible replicates belonging to a particular individual. In this way, each group of sub-samples always encompasses the same number of individuals but with different randomly chosen representative replicates. We generated a large enough number of replicated models (e.g., around 1000) with subsequent resulting ranking lists from each of them, which were then combined (aggregated) in one final list with a more reliable features ranking list.

The contribution of cumulative ranked feature indices was obtained by the cardinality of each feature index in the dataset from sub-lists consisting of the k ranked element from each of the generated lists in the set (as defined in [47]) (Figure S1, Supplementary Material), where k was given for three different intervals: (a) 1 < k < 5, (b) 6 < k < 10, and (c) 11 < k < 15. According to the results for relevant features (Figure 3), the first 30 features from the obtained variable importance list represent the most relevant features for each generated Random Forest variable importance model. As can be seen from Figure S1a, features “*m*/*z* 293.1779”, “Cer 36:2;O3”, “Cer 34:2;O2” and “Cer 34:1;O2 A” had almost the same values for extraction numbers (frequency counts) for each segment of the first five positions for the total set of ranking sub-lists. Therefore, each of them could be evenly ranked to any of the first four positions of the general ranking list. Features like “LPC 18:2 A”, “FA 16:1”, “FA 18:2”, “FA 16:0”, “SM 34:2;O2 A1”, “Cer 36:0;O2”, although they have a relatively low extraction number in the first five ranking positions, gradually gained relevance through increasing extraction numbers in the next two segments of the cumulative ranking lists (each encompasses five successive ranking positions in each sub-list; Figure S1b,c in Supplementary Material). A summary overview for the cumulative frequency count for the first 30 positions in the sub-list set of generated ranking lists is presented in Figure S2. All features possessing the highest possible counts (23 of 30) could be ranked in the general ranking list among any of the first 30 positions. However, for more precise ranking of all features at certain positions, different kinds of aggregation were applied.

The most straightforward way to aggregate the N different ranked lists was to use the Borda count method [47], an algorithm well known in voting theory that assigns a score equal to the sum of the number of features with higher position over the N lists to each variable. Then, the global ranked list was obtained by ordering the features according to the Borda count [54,55]. The Borda count method is equivalent to ranking the features according to their average rank obtained over the N lists. Applying the Borda count method to 1000 generated ranking lists obtained through the RF classifier provided was performed with the resulting aggregating ranked list (Table S1).

**Figure 4:**
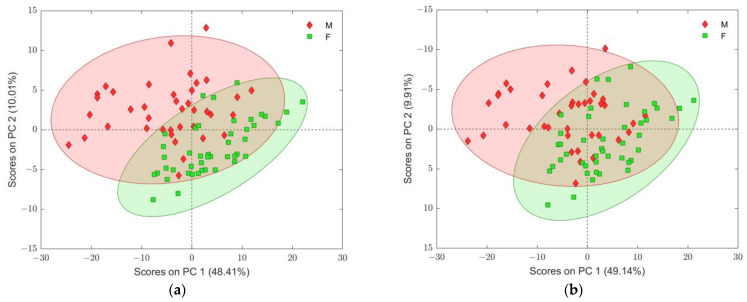
(**a**) PCA score plot of C group of samples after removal of the first 15 ranked variables given in Table S1; (**b**) PCA score plot of C group of samples after removal of the first 30 ranked variables given in Table S1; samples are assigned according to gender, where the confidence level of Hotelling’s T2 ellipses (rounding each sub-class) was 95% in each plot. Sub-classes were assigned to gender identity (males: M with red diamonds and females: F with green squares).

To examine the influence of ranked features on overall variability in the control group of the dataset (two PCA models were composed), the first 15 ranked features (Table S1) were excluded from the dataset (Figure 4a) in the first one, and then the next 15 features (overall the first 30 features) were excluded for the second PCA model (Figure 4b). As can be seen from both depicted PCA score plots, separation between genders’ sub-classes gradually diminished, indicating the significant importance of the first 30 variables in the resulting ranked list. However, from the same score plots, it could also be noticed that other variables with higher ranking positions still have a certain influence on sub-class separation in the presented PCA models.

Therefore, during supervised OPLS-DA classification, it is more appropriate to use a separate dataset, where each has the same gender class membership. In other words, one extracted dataset including all males inside the Control and Schizophrenia classes, and another one with all females, also comprising the main classes (C and SCH), were used for the forthcoming supervised classification.

### 2.3. OPLS-DA Models

The OPLS-DA regression algorithm is a supervised classification technique [27,28,56,57,58] that enables discrimination between diverse classes of samples and, at the same time, identification of statistically relevant compounds/metabolites responsible for such discrimination between class of ‘schizophrenia’ patient in comparison to the representative group of non-psychiatric controls.

After exclusion of identified outliers, the overall dataset was divided according to gender affiliation into two subsets: one with all male constituents (containing 39 observations of 14 individuals from the control group and 44 observations from 15 schizophrenia patients, overall 83 samples), and another with all female constituents (including 45 observations of 15 individuals from the control group and 41 observation from 14 schizophrenia patients, overall 86 samples). For the validation purpose of the model, datasets for each of the gender affiliations were partitioned independently into five disjointed subsets or folds (comprising approximately even numbers of samples), where the structures of triplicates belonging to each sample were preserved. Each of the partitioned subsets represented an independent test set for the particular fold, while the remaining four subsets in the same fold were arranged together to form the calibration or training dataset. In this way, five sets for calibration and prediction purpose for OPLS-DA classification for each of the gender affiliations were assembled. In addition, each of four groups of any of the calibration sets could be utilized for cross-validation purpose during OPLS-DA model training.

For evaluation of the classification performance of the applied classification models, different diagnostic statistics (accuracy, misclassification rate, sensitivity, and specificity) were used for this purpose in metabolomics when PLS-DA models were applied [59,60]. However, in situations when the classifier showed almost perfect classification performance, such measures of model performance could be less informative. Since the PLS-DA classification primarily performed regression between the matrix of observation and class membership variable, the performance of the cross-validated calibration model could also be estimated through the R_cv_^2^ and RMSECV, provided through the same model outputs. In addition, the prediction performance could also be measured in terms of R_p_^2^ and RMSEP. For good performance of the model terms, R_cv_^2^ and R_p_^2^ were expected to be close to 0.9, while the root mean square error terms should be close to zero [61,62]. Besides, confusion matrices for the calibration model and prediction results were observed in all cases. The accuracy and misclassification rate of classification were also determined from these confusion matrices.

### 2.4. Male Subset of Samples

Results of the cross-validation and prediction for calibration and validation test datasets of the male dataset are presented in Table 1.

Each model (Table 1) was optimized according to critical parameters for both calibration and prediction subsets used in modeling. As result, the optimal number of components was chosen to satisfy the optimal value for RMSECV, RMSEP, R_cv_^2^, R_p_^2^ and Accuracy for both calibration and prediction datasets. The resulting number of features obtained according to VIP score values higher than 1 is given in Table 1.

All prediction values (Table 1) for accuracy and misclassification rate were indicative for three almost perfect classifications from the test datasets, except for the training and test datasets assigned to subset 4. The training dataset showed perfect classification while, at the same time, the prediction test data subset showed an accuracy of 0.8333, indicating somewhat lower model classification performance in prediction. These results were indicative of the presence of misleading samples in the test dataset, which were missing from the corresponding calibration dataset. Because of the nature of partitioning of the initial dataset (containing all male constituents), these potentially misleading samples were almost certainly present in all other calibration sets (folds: 1, 2, 3 and 5) explaining the observed (poor) classification performance and subsequently good prediction performance of the test data subset for corresponding models.

It should be emphasized that values for RMSECV and R_cv_^2^, as well as RMSEP and R_p_^2^, were inversely correlated for all data in Table 1 where, at the same time, values for R_cv_^2^ and accuracy were directly and inversely correlated with corresponding values of the RMSECV. These were a good indication for later comparison of OPLS-DA models composed for the female dataset.

**Figure 5:**
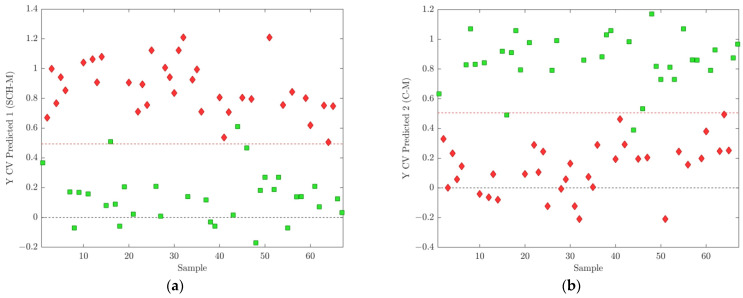
(**a**) Cross-validated prediction results for class SCH-M, where the discrimination threshold was determined at 0.4943 (red dashed line); (**b**) cross validated prediction results for class C-M, where the discrimination threshold was determined at 0.5057 (red dashed line). The schizophrenia cohort is shown in red diamonds, and the control group in green squares.

**Figure 6:**
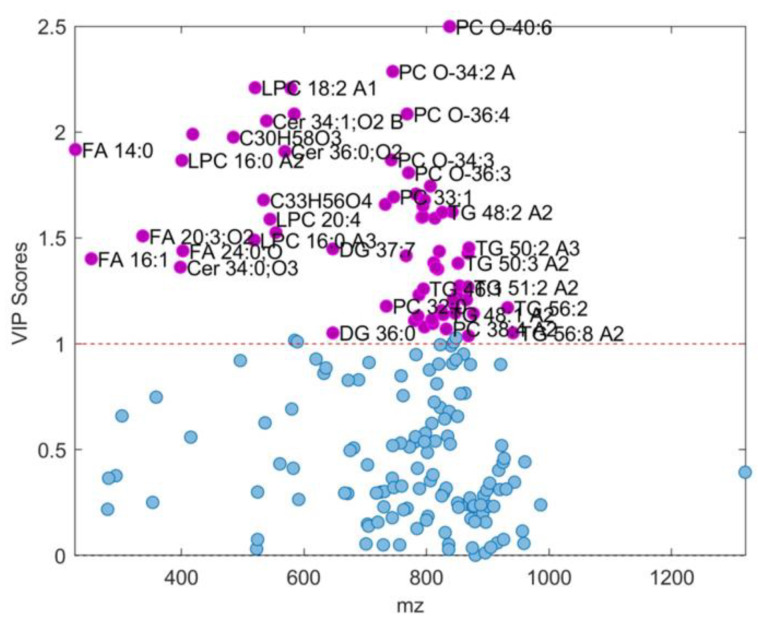
VIP scores > 1 (shown as purple dots accompanied with appropriate feature assignment for each (see Table 2 for details) determined from OPLS-DA model composed from male subset assigned as fold 3 with accuracy of 0.9701 (see Table 1). LPC: 1-acyl-sn-glycero-3-phosphocholines; FA: fatty acids; Cer: *N*-acylsphinganines (dihydroceramides); PC: diacylglycerolphospho-cholines; PC O-: 1-alkyl,2-acylglycerophosphocholines; DG: diacylglycerols; TG: triacylglycerols. Features with a VIP score < 1 are shown as blue dots.

**Figure 7:**
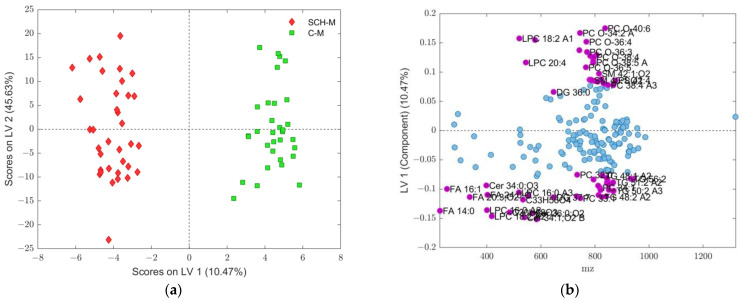
(**a**) Score plot of OPLS-DA model composed from data subset assigned to fold 3 in Table 1, where discrimination classes belong to C-M (green squares) and SCH-M (red diamonds) groups of samples; (**b**) loadings of LV1 component, where assigned features, shown as purple dots (see Table 2 for details), were obtained according to VIP scores > 1 from OPLS-DA, while features with a VIP score < 1 are shown as blue dots. LPC: 1-acyl-sn-glycero-3-phosphocholines; FA: fatty acids; Cer: *N*-acylsphinganines (dihydroceramides); SM: ceramide phosphocholines (sphingomyelins); PC: diacylglycerolphospho-cholines; PC O-: 1-alkyl,2-acylglycerophosphocholines; DG: diacylglycerols; TG: triacylglycerols.

The best model among all five partitioned datasets was the model for subset 3. This showed a good balance of all performance-compared parameters and the highest accuracy of 0.9701. Corresponding results for OPLS-DA models obtained for fold 3 subdivision are presented in Figure 5, Figure 6 and Figure 7 and Table 2. According to this model, 69 differential *m*/*z* features belonging to the 49 molecules (Figure 6 and Figure 7b and Table 2) showed the highest contributions to group separation, with VIP values higher than 1.0.

### 2.5. Female Subset of Samples

Results of the cross-validated and predicted calibration and validated test datasets for female dataset showed perfect classification for all composed models (accuracy was 1.0 for calibration and test models) (Table 3). Gradation in model performance in such cases could only be performed according to RMSECV, R_cv_^2^ (CV) in calibration and RMSEP, and R_p_^2^ in prediction for observed models. In this respect, models belonging to fold 1 showed the best performance, with RMSECV = 0.1635 accompanied with RMSEP = 0.0818 for prediction.

**Figure 8:**
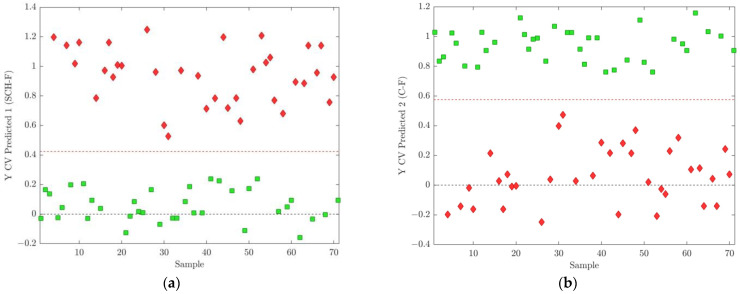
(**a**) Cross-validated prediction results for class SCH-F, where the discrimination threshold was determined at 0.4242 (red dashed line); (**b**) cross-validated prediction results for class C-F, where the discrimination threshold was determined at 0.5758 (red dashed line). The schizophrenia cohort is shown in red diamonds, and the control group in green squares.

**Figure 9:**
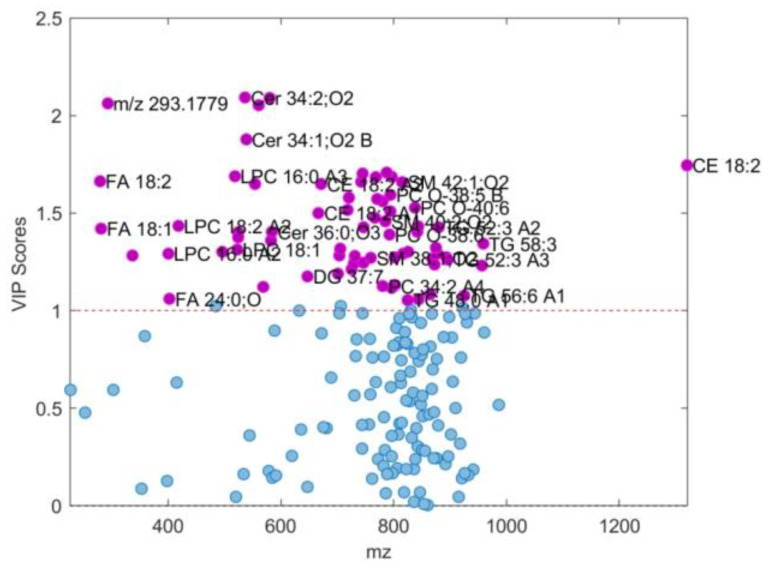
VIP scores > 1 (shown as purple dots accompanied with appropriate feature assignment for each (see Table 4 for details) determined from OPLS-DA model composed of female subset assigned as fold 1, which gives the best RMSEP = 0.0818 and R_p_^2^ = 0.9767 for prediction (see Table 3). LPC: 1-acyl-sn-glycero-3-phosphocholines; FA: fatty acids; Cer: *N*-acylsphinganines (dihydroceramides); PC: diacylglycerolphospho-cholines; PC O-: 1-alkyl,2-acylglycerophosphocholines; SM: ceramide phosphocholines (sphingomyelins); DG: diacylglycerols; TG: triacylglycerols; CE: cholesterol esters. Features with a VIP score < 1 are shown as blue dots.

**Figure 10:**
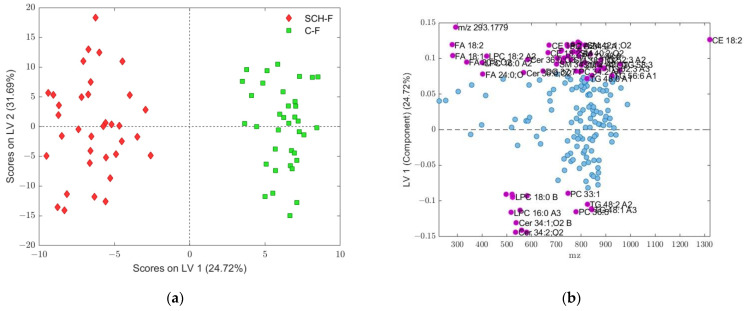
(**a**) Score plot of OPLS-DA model composed from data subset assigned to fold 1 given in Table 3, where discrimination classes belong to C-F (green squares) and SCH-F (red diamonds) groups of samples; (**b**) loadings of LV1 component, where assigned features, shown as purple dots (see Table 4 for details), were obtained according to VIP scores > 1, while features with a VIP score < 1 are shown as blue dots. LPC: 1-acyl-sn-glycero-3-phosphocholines; FA: fatty acids; Cer: *N*-acylsphinganines (dihydroceramides); PC: diacylglycerolphospho-cholines; SM: ceramide phosphocholines (sphingomyelins); DG: diacylglycerols; TG: triacylglycerols; CE: cholesterol esters.

According to the corresponding results for OPLS-DA models obtained for the fold 1 subdivision of the original dataset including all female participants (Figure 8, Figure 9 and Figure 10, and Table 4), 74 differential *m*/*z* features belonging to the 60 molecules (Figure 9 and Figure 10b, and Table 4) showed the highest contributions to group separation, with VIP values higher than 1.0.

### 2.6. Relevant Feature Validation

VIP (variable importance in projection) was used for selection of relevant feature subsets [63], for which less complex OPLS-DA models should be expected in comparison with full dataset OPLS-DA models. One of the critical steps in the model development scheme, which requires extensive validation, is predictor variable selection [64]. Variable selection is usually conducted to improve some statistical parameter (such as R^2^Y or Q^2^Y) or simply to achieve model simplification. However, in a situation where the dataset under investigation has a limited number of samples (as in this study), dividing the dataset (into calibration and test partition) to perform external validation is not feasible, and in these cases a permutation test is recommended as a valuable addition to the internal validation scheme [64].

In the previous section, details of the conducted analysis were specified, where the dataset for each gender sub-class was divided into five folds, utilized to obtain five independent calibration and prediction datasets. For each of these, OPLS-DA model’s parameters were developed and optimized, where the details regarding the obtained results have already been discussed. By comparison of the obtained results and the related diagnostic statistics used to measure performance among models for each of the folded groups of the calibration and related testing/prediction models, some inconstancy could be observed among output results. For VIP scores higher than 1 (Table 1) for male subjects, containing a set of selected features in the range between 60 and 69, the best model according to performance measures was estimated to be a subset of the fold 3 group. On the other hand, a similar comparison of VIP scores (higher than 1) for female subjects presented (Table 3) provided sets of selected features ranging from 74 to 83, where the best model for this sub-class, according to obtained statistics, belonged to a subset of fold 1. Furthermore, each set of VIP scores contained a different number of features, where the features within each group vary in terms of both number and order. Subsequently, the estimated optimal number of latent variables (components) used for composing models was four for the male part of the dataset and two for the female part of dataset. Therefore, to improve consistency across various feature lists when assembling the OPLS-DA model, further validation of the acquired features should be carried out in the proceeding section.

The complete procedure used to accomplish feature validation analysis, with details regarding the algorithm utilized for this purpose, including obtained results, is presented in the Supplementary Material under sections titled *Feature validation* and *Permutation test* [55,59,64,65,66] (Figures S3–S6).

According to the obtained results for the dataset containing only male individuals, subsets with 41 ranking features were considered relevant. Likewise, in similar findings for a dataset that exclusively contains female participants, the best subset should contain 61 ranked features. Among identified features, in both gender subsets, there were 23 shared features (common to both gender subsets), which could be regarded as relevant for overall class separation. Validated relevant features identified in the present analysis (both for males in Table 2, and females in Table 4, and for both groups in Supplementary Material Table S1), were distinguished by a hash sign among previously identified metabolites. Comparison of the ranking lists obtained from VIP scores higher than 1 from male subjects (fold 3 subset presented in Table 2) and the subset of the first 41 ranked variables obtained as a result of validation, showed very close matching (only two features are different). Similar comparison for female subjects (fold 1 subset presented in Table 4) and the subset of the first 61 ranked variables from the obtained results for validation also showed very good agreement (only four features are different). Such analysis also suggested that obtained ranking lists, according to the 5-fold partitioned dataset presented in the previous section and obtained according to VIP score values higher than 1, were relevant to some extent and in some instances, such as in the present work, though additional validation should be performed. In addition, conducted Permutation tests (Supplementary Materials section *Permutation test*) for OPLS-DA models assembled with a complete set and selected subsets of features confirmed that all considered diagnostic measures of performance proved to be statistically significant (*p* < 0.001). A considerable improvement in diagnostic statistics was observed by comparison of real unpermuted models with all included features and models with only a selected number of features.

## 3. Discussion

In recent years, several papers on plasma and serum lipidomics studies of SCH, applying LC coupled to HR-MS, have been published [19,36,37,38,39,40,41]. Compared to the PCA plots of all samples that showed no clear separation between the SCH and C groups (Figure 1), PCA results using a gender variable to assign sub-classes to both groups indicated a gender separation only within the C group (C-M and C-F) (Figure 2). VIP values along with other metrics (Figure 3, Figure 4, Figures S1 and S2 and Table 2 and Table 4) indicated that the putatively identified lipids performed well in the gender differentiation of C-M and C-F groups, which is also supported by literature data [67,68,69,70]. Our results demonstrated differentiation of five main lipid classes in control C-M and C-F groups: glycerophospholipids (PC, PS, PC-O, LPC), sphingolipids (Cer, SM), glycerolipids (DG, TG), fatty acids (FA) and cholesterol esters (CE) (Table S1). The gender effect on the blood lipidome in three large population level studies, the Alzheimer’s disease neuroimaging initiative—ADNI (n = 806), the GeneBank Functional Cardio-Metabolomics cohort (n = 1015), and the Genetics of Lipid lowering Drugs and Diet Network—GOLDN (n = 422), confirmed that about 60% of lipids distinguish between men and women in all three cohorts and showed that the content of phosphatidylcholines (PC), phosphatidylethanolamines (PE), phosphatidylinositols (PI), Cer, SM and CE is higher in females, while the content of TG and LPC is higher in males across the three cohorts [67]. Ishikawa et al. (2014) also pointed out the gender-associated differences in lipid profiles, emphasizing remarkably higher levels of many sphingomyelins in females, irrespective of age and matrix (plasma and serum) [68]. Slade et al. (2021) explored lipidomic profiles of 980 participants aged 18–87 years old from the Genetics of Lipid-Lowering Drugs and Diet Network (GOLDN) and concluded that sex effects on lipidomics are most prevalent among PC, SM and TG [69]. Tabassum et al. (2023) also emphasized the potential influence of genetic factors, namely sex chromosomes and sex-specific physiological factors, such as menopause and sex hormones, to the gender-associated differences in lipidomic profiles of men and women [70]. Obviously, all these literature data highlighted that the mechanism of regulation of lipid metabolism might be different between men and women and lipidomics of blood samples (serum and plasma) should be discussed for male and female samples separately, treating gender as a distract factor [67,68,69,70].

Therefore, our further chemometric analysis and results (OPLS-models, CV prediction results, VIP values) are based on investigation and comparison of (1) lipid profiles of male schizophrenia patients SCH-M and male non-psychiatric controls C-M and (2) lipid profiles of female schizophrenia patients SCH-F and female non-psychiatric controls C-F, as well as analysis of differential lipids in these two groups (Figure 5, Figure 6, Figure 7, Figure 8, Figure 9 and Figure 10, Table 2 and Table 4). It is worth mentioning that up to now only one paper has been found that studied and compared lipidomics profiling of gender-separated samples, i.e., specifically between plasma SCH-M samples and male controls C-M [71].

Analysis of the obtained results using OPLS-models of SCH-M and C-M groups showed that forty-nine lipid molecules contributed to group separation with VIP values higher than 1.0 (Figure 6 and Figure 7 and Table 2). These lipids belong to four main lipid classes: GP (PC, PS, PA, PC-O, LPC), SP (Cer, SM), GL (DG, TG) and FA (saturated and unsaturated fatty acids) (Figure 7 and Table 2) and most of these lipids had a higher content in the controls C-M than in the SCH-M group, with LV1 values higher than 0.1 (Figure 7b). There were 17 lipid molecules that showed higher contents in SCH-M compared to C group (LV1 < −0.1) including Cer 36:2;O3, Cer 34:1;O2 and Cer 36:0;O2, SM 42:2;O2, PC 33:1 and PC 32:1, LPC 18:2 and LPC 16:0, DG 37:7,TG 48:2, TG 50:3 and TG 50:2,FA 14:0, FA 20:3 and FA 24:0 and two unidentified molecules (C30H58O3 and C33H56O4) (Figure 7b and Table 2).

On the other hand, OPLS-models of SCH-F and C-F groups confirmed sixty differential lipid molecules identified to be significant for discriminating SCH-F patients from non-psychiatric controls C-F, including five lipid classes: GP (PC, PC-O, LPC), SP (Cer, SM), GLDG, TG, FA (saturated and unsaturated fatty acids) and (CE) (Figure 8, Figure 9 and Figure 10 and Table 4). There were eight lipid molecules that had a higher content in SCH-F compared to C-F group (LV1 < −0.1) including Cer 36:2;O3, Cer 34:1;O2, A, Cer 34:2;O2 and Cer 34:1;O2 B), PC 36:5), LPC 16:0) and TG 48:1 and TG 48:2 (Figure 10b and Table 4). Most of these differential lipids had a higher content in the control C-F than in the SCH-F group with LV1 values higher than 0.1 (Figure 10b and Table 4), similar when compared to the differences found for SCH-M and C-M groups (Figure 7b and Table 2). From differential lipid metabolites with higher content in SCH-F compared to C-F group, as well as in SCH-M compared to C-M group, there were also four common lipid molecules: Cer 34:2;O3 and Cer 34:1;O2 A, LPC 16:0 and TG 48:2 (Figure 7b and Figure 10b).

Tkachev et al. (2021) determined the abundance of Cer(d18:1/16:0), Cer(d18:1/18:0) and Cer(d18:1/24:1) in the plasma of 82 SCH patients and 138 controls using LC–HRMS and confirmed a higher content of all three types of Cer in patients with SCH compared to control groups, which is in agreement with our results for Cer 34:1 in both SCH groups [37]. Costa et al. (2023) explored untargeted lipidomics of plasma samples from drug-naïve patients with SCH in comparison to healthy controls and found a higher mean intensity of Cer 44:2 and Cer 42:2, while mainly ceramides had lower intensity than controls [19], which is opposite to our results. Li et al. (2022) studied the erythrocyte membrane lipidome in SCH and healthy controls showing that FA 16:0, FA 18:0 and FA 18:1 have significantly increased abundance in ceramides of SCH than control group, indicating good agreement with our results [72]. Literature data also indicated that SMs with monounsaturated fatty acids MUFAs (FA 18:1 and FA 24:1) were increased in plasma of the SCH patients compared to controls [19], as well as in our results for SCH-M, while SMs with saturated fatty acids SFAs (FA 16:0, FA 20:0, and FA 24:0) [39] and most of the membrane SMs were decreased [72], as in our results for both SCH-M and SCH-F compared to C-M and C-F, respectively (Figure 7b and Figure 10b, and Table 2 and Table 4). Lipids have a very important function in the development of SCH pathogenesis [72]. Alteration in phospholipids content is correlated with disruption of neurochemical parameters (dopamine and glutamate) [73]. SMs and Cers show an influence on the presynaptic release of dopamine [74]. Increased Cer concentrations are correlated with progression of depression because they reduce dopamine transport by affecting the dopamine transporter function and cause an increase in serotonin transport [75].

In our SCH-M group from phosphatidylcholines (PC and PC-O), most have lower content compared to controls C-M, as well as in most of the available literature data [19,36,38,39,40,41,72,76,77,78], except for PC 33:1 and PC 32:1, which are up-regulated and matched with the results of Li et al. (2022) [72]. Mostly, all LPCs (LPC 18:2 and LPC 16:0) had significantly higher content in SCH-M than in C-M, and in one LPC 16:0 in SCH-F compared to C-F, which agrees with the obtained results of Costa’s and Li’s research groups [19,72].

Phospholipid metabolism abnormalities are closely connected with an increase in phospholipase A2 (PLA2) activity, which catalyzes the hydrolysis of unsaturated FA from the sn-2 position of the glycerol moiety of GP. In membranes of the neural system, upregulation of the phospholipase A2 is correlated with the dopamine system, causing polyunsaturated fatty acid (PUFA) dissociation and SFA incorporation in membrane phospholipids. Decomposition of membrane phospholipids causes a decrease in synthesis of PCs and conversion of PC-containing linoleic acid into AA and, subsequently, AA transformation into pro-inflammatory prostaglandins (PGs) [75], which are responsible for neuroinflammation and oxidative stress. Increased levels of LPC 16:0, lysophosphatidyl serine LPS 18:0, and SM 24:0, were also closely correlated with inflammation and oxidative stress. Chronic stress triggers the hypothalamic–pituitary–adrenal axis (HPA) hyperactivity, increased levels of glucocorticoids and phospholipase D activity [75]. Increased phospholipase D activity causes conversion of PC and PE into PA, as well as choline and ethanolamine, respectively, and PA can be further converted into DG. Increased levels of DG, LPC and lysophosphatydilethanolamine (LPE) influence membrane destabilization and higher glucocorticoids concentrations in the cells, as well as decreased triacylglycerol hydrolase activity and increased TG synthesis activated by higher diacylglycerol acyltransferase 2 activity. Most lipidomic results for the content of DG and TG in SCH patient samples indicated that their levels were higher compared to controls, while in our results the content of only one DG (DG 37:7) and three TGs (TG 48:2, TG 50:3 and TG 50:2) was higher in SCH-M samples compared to C-M, and of one TG 48:2 in SCH-F compared to C-F group [37,38,72,79,80,81]. The contents of most of the other identified DGs and TGs were lower for both SCH-M and SCH-F groups.

Our results of the lipid profiles study of gender-differentiated groups, between SCH-M and C-M, as well as between SCH-F and C-F groups, indicate that the alteration of lipid metabolism in SCH could be tightly associated with the modulation or enrichment of enzyme PLA2 activity. PLA2 has a catalytic role in the decomposition of GP into corresponding, usually unsaturated, FA and lysophospholipid. The increased PLA2 activity has already been reported in SCH, and its close relation with alteration in neuronal function, which influences affective and cognitive symptoms, was emphasized [19]. Putative lipid biomarkers determined in our lipidomics study, especially metabolites that were common for both sex-differentiated SCH-M and SCH-F groups compared to their healthy controls, indicate the importance of enzymes involved in the regulation of their content, as well as of themselves, in further research into a universal set of biomarkers, as targets for SCH diagnosis and treatment.

Finally, our study also has some limitations. Firstly, a total of 61 participants were included in this study, and this moderate sample size can lead to a limitation of statistical power. Secondly, the obtained results were not achieved by a fully quantitative and validated method, and potential biomarkers were not approved by applying reference material. Thirdly, although sex-differences were included in our study, other factors that should be considered are age-differences, as well as dietary habits and application of antipsychotics of the first and the second generation, because these might have influence on the content of GPs and SPs [36].

## 4. Materials and Methods

### 4.1. Sampling

This study was performed in accordance with the Ethics Committee of the Special Hospital for Psychiatric Diseases “Kovin”, the University of Belgrade—Faculty of Chemistry, and the Blood Transfusion Institute of Serbia. Patients’ blood samples were obtained from the Special Hospital for Psychiatric Diseases “Kovin”, while samples of non-psychiatric healthy controls were provided by the Blood Transfusion Institute. All participants or their caretakers provided written consent before their enrollment in this study. A total of 30 SCH patients and 31 non-psychiatric healthy volunteers (controls) were involved in this study. There were no statistically significant differences in age (with ages between 24 and 74 years), gender (15 SCH-M, 15 SCH-F, 16 C-M and 15 C-F), or BMI (20.05–38.44 kg/m^2^) between the patient and control groups. All patients (100%) were using anxiolytics (clonazepam, diazepam, lorazepam). In addition, 2 patients (6.7%) were using antipsychotics of the first generation (chlorpromazine, fluphenazine, haloperidol, levomepromazine), 15 patients (50.0%) were using antipsychotics of the second generation (aripiprazole, clozapine, quetiapine, olanzapine, risperidone), and 13 patients (43.3%) were using both, antipsychotics of the first and the second generation. Healthy controls were under no medical therapy. The SCH patients and non-psychiatric controls followed a typical, traditional eating pattern that seems to be associated with high intake of fat, SFA, and low of n-3 PUFA and could have an unfavorably high n-6/n-3 PUFA ratio [82,83]. Blood samples were collected from the patients and non-psychiatric healthy controls in the morning hours, before the first meal and after a minimum 8 h of fasting.

### 4.2. Sample Preparation

The blood samples were kept on ice for one hour and centrifuged. The sera collected from the supernatants were stored at −80 °C. The maximum period of storage before analysis was up to two weeks. Lipid extraction from serum samples was performed in triplicate, according to the procedure given by O’Brien et al. (2019) [84]. LC–HRMS measurements, LC–HRMS data processing and statistical analysis (with slight modifications) were performed as described in Jadranin et al. (2023) [32] and can be found in the Supplementary Material.

### 4.3. Readings in Data

The resulting table of retention times, *m*/*z* values, and peak intensities was organized into a single matrix containing the samples (cases) in the rows and the *m*/*z* (rt) values in the columns (variables). Additional categorical variables relating to the main class affiliation of the samples (class variable: SCH—schizophrenia patients and C—non-psychiatric healthy controls), gender identity (M—for male and F—for female), and triplicate grouping identifiers, were also settled in the final dataset structure, in a similar manner as in our previous work [27,28,32]. A replicated sample in this work was primarily used in the sense of identifying different acquired LCMS chromatograms originated from the same sample of individuals. The initial dataset matrix was constructed with 183 chromatograms (30 patients organized in 90 triplicates for the SCH group, and 31 individuals organized in 93 triplicates for the C group of dataset) and 192 variables for combined negative–positive ions.

### 4.4. Software

Data processing and chemometric analysis in this work was accomplished using toolboxes and software implementations, including in-house developed routines, conducted under MATLAB version 9.7 (MathWorks Natick, MA, USA) [85]. In some instances, appropriate packages developed under R environment [86] were also additionally exploited (pmartR [87], malbacR [88], “randomForest” [52], “ranger” [53], “votesys” [54]). Routine for the borda count method was utilized from python module mlpy [55]. Reading in LCMS spectra and chromatograms into MATLAB workspace was realized via R package XCMS version 3.22.0 [89]. Beside classical PCA analysis, for the purpose of efficiently outlier detection, a robust PCA (ROBPCA) method [42,43] as a part of LIBRA Matlab package [44] was also used. Pre-processing and chemometrics analysis of selected LCMS data were performed by PLS Toolbox version 8.9.1 [90].

### 4.5. Data Pre-Treatment

Before chemometric modelling and processing, the potential effects that various data pre-treatments (handling of missing data (if such exist), data transforming, normalization and finally centering and scaling) may have on the distribution of data and, in turn, the outcomes of chemometric modeling have been analyzed, at first individually, and then in conjunction with other mentioned pre-processing techniques.

The missing values were initially estimated by the column-wise median value as a part of the ROBPCA function [42]. In addition, we have tested an imputation method using the “movemedian” option, where missing values were replaced with median over a window of three data points length around the missing value. However, replacing missing data with linear interpolation of neighboring, non-missing values was shown as most suitable for the dataset under investigation [91]. As a result, all missing values identified in our dataset were handled using the linear interpolation method.

To ensure producing statistically meaningful metabolomic data, logarithmic transformation of data (i.e., replacing each value, x, with log10(x) or log2(x)) [92,93] was performed. This had the effect of monotonically reducing extremely high values, which in turn produces homoscedastic and near-normal or near-Gaussian model residuals. The outcome from boxplot of the log10-transformed LC–HRMS dataset is presented in Figure S7a (Supplementary Material), where grouping was performed along both main classes, SCH and C, and the boxplot of the same data, additionally mean centered after logarithmic transformation, is presented in Figure S7b (Supplementary Material). Results of log10 transformation presented in Figure S7a indicate that skewness of data was significantly reduced in comparison with untransformed data. However, results presented in Figure S7b also suggested that mean centering and subsequent scaling to (i.e., dividing of each variable with) the standard deviation of corresponding variables (i.e., autoscaling) would be most beneficial before any further modelling.

Given that PCA analysis of samples analyzed in four consecutive batches (Figure S8, Supplementary Material) did not show a significant drift, and that, when comparing intra-day and inter-day coefficients of variation (CV) for filtered *m*/*z* (rt) values in the QC samples, CV was below 30%, variations in instrument sensitivity during the measurements were considered not to affect the results, so there was no need for further normalization of the samples. Therefore, automatic scaling after log transformation (including mean centering and scaling to the standard deviation of each variable) was used for data pre-treatment before further processing (PCA or OPLS-DA modelling).

### 4.6. Outlier Detection

The diagnostic outlier map plot obtained from “robpca” Matlab routine [42,43,44], which was based on the score distances and orthogonal distances computed for each observation (see Figure S9), has been used to identify outlying observations (the procedure is explained in detail in the Supplementary Material [42,94,95]).

In that way, 14 distinctive outliers have been isolated from the original dataset, so 192 *m*/*z* features for combined negative–positive ion modes, obtained by using the LC–HRMS method, and 169 chromatograms were included in the final dataset and subjected to the multivariate statistical analyses (PCA and OPLS-DA).

### 4.7. Lipids Annotation

The lipids were tentatively assigned using the accurate mass measurements and databases—LIPID MAPS Structure Database (LMSD) (https://www.lipidmaps.org/, accessed on 12 July 2024) [96] and Human Metabolome Database (HMDB) (https://hmdb.ca/, accessed on 12 July 2024) [97].

## 5. Conclusions

Alteration of GP, SP and GL metabolism showed an important role of lipid pathways in the pathogenesis of schizophrenia. The MS-based untargeted lipidomics study of gender-differentiated groups, between SCH-M and C-M, as well as between SCH-F and C-F groups, confirmed that lipids metabolism was definitively altered, with decreased content of the majority of the most affected lipid classes: GP, SP, GL, FA and CE. GPs, SPs and GLs are the most abundant among differential lipids. From differential lipid metabolites with higher content in both SCH-M and SCH-F patients groups compared to non-psychiatric controls, there are also four common lipid molecules: Cer 34:2 and Cer 34:1, LPC 16:0 and TG 48:2. Increased concentrations of Cer induce increase in activity of PLA2, catalyzing the hydrolysis of GP into FAs, which can be further transformed into other lipids, including pro-inflammatory lipid mediators. Increased levels of LPC influence on membrane destabilization and decreased triacylglycerol hydrolase activity cause increased TG synthesis activated by higher diacylglycerol acyltransferase 2 activity.

With aim of accomplishing a universal set of biomarkers, it is necessary to explore a comprehensive analysis of serum and plasma samples of SCH patients, including larger sample sizes, sex-differences, age-differences, diet habit and application of antipsychotics, to remove doubts about the influence of all these factors on lipid pathways. Validation of differential lipids, including Cer 34:2, Cer 34:1, LPC 16:0. TG 48:2, inflammatory factors and neurotransmitters applying targeted lipidomics is also necessary to study the biochemical alterations in SCH lipid metabolism and to clarify and understand lipid pathways and their application in SCH diagnosis.

## Figures and Tables

**Table 1:** Classification performance for the OPLS-DA models obtained for each subset of male samples.

Fold Number	*n* Comp	RMSECV	RMSEP	R_cv_^2^	R_p_^2^	VIP Selected	Calibration		Prediction	
							Accuracy	Misclass. Rate	Accuracy	Misclass. Rate
1	3	0.2729	0.1804	0.7123	0.9312	61	0.9412	0.0588	1	0
2	3	0.2606	0.1906	0.7345	0.8752	60	0.9538	0.0462	1	0
3	4	0.2198	0.2518	0.8162	0.7540	69	0.9701	0.0299	1	0
4	4	0.1947	0.3142	0.8529	0.6319	64	1.0000	0.0000	0.8333	0.1667
5	4	0.2427	0.1572	0.7682	0.9100	66	0.9552	0.0448	1	0

The cross-validation was performed for each training subset, and model quality was evaluated by RMSECV, R_cv_^2^ (CV) and accuracy and misclassification rate for calibration. Prediction performance measure was given through RMSEP, R_p_^2^, and accuracy and misclassification rate for prediction. Number of selected variables was assigned according to VIP scores > 1 in all cases. n—Number of components, RMSECV—root mean square error of cross-validation, RMSEP—root mean square error of prediction, R_cv_^2^—correlation coefficient of cross-validation, CV—cross-validation, R_p_^2^—correlation coefficient of prediction, VIP—variable importance in projection.

**Table 2:** Relevant *m*/*z* values according to the OPLS-DA model (combined positive and negative ion modes) relating to the lipids found differently in male schizophrenia patients (SCH-M) and healthy controls (C-M).

No.	Retention Time (min)	VIP Value	VIP Feature Assignment	Measured *m*/*z* *	Ion Mode Adduct	Proposed Formula	Lipid Assignment	Lipid Class
1	1.26	1.5893	LPC 20:4	544.3385	[M+H]^+^	C_28_H_50_NO_7_P	LPC 20:4	GP
2	1.29	2.2064	LPC 18:2 A ^#^	578.3494	[M+OAc]^−^	C_26_H_50_NO_7_P	LPC 18:2	GP
		2.2096	LPC 18:2 A1 ^#^	520.3385	[M+H]^+^			
		1.9903	**LPC 18:2 A2** ^#^	542.3228	[M+Na]^+^			
3	1.52	1.5258	**LPC 16:0 A** ^#^	554.3496	[M+OAc]^−^	C_24_H_50_NO_7_P	LPC 16:0	GP
		1.8664	**LPC 16:0 A2** ^#^	513.3667	[M+NH_4_]^+^			
		1.4902	**LPC 16:0 A3** ^#^	518.3202	[M+Na]^+^			
4	1.76	1.9168	**FA 14:0** ^#^	227.2027	[M−H]^−^	C_14_H_28_O_2_	FA 14:0	FA
5	2.04	1.4009	FA 16:1 ^#^	253.2187	[M−H]^−^	C_16_H_30_O_2_	FA 16:1	FA
6	2.15	1.4385	**FA 24:0;O** ^#^	402.3924	[M+NH_4_]^+^	C_24_H_28_O_3_	FA 24:0;O	FA
7	2.80	1.6790	**C33H56O4** ^#^	534.4503	[M+NH_4_]^+^	C_33_H_56_O_4_	N.D.	N.D.
8	2.89	1.5088	**FA 20:3;O2** ^#^	337.2388	[M−H]^−^	C_20_H_34_O_4_	FA 20:3;O2	FA
9	3.12	1.0098	PA 25:0	589.3263	[M+K]^+^	C_28_H_55_O_8_P	PA 25:0	GP
10	4.52	1.9748	**C30H58O3** ^#^	484.4709	[M+H]^+^	C_30_H_58_O_3_	N.D.	N.D.
11	5.02	1.3615	Cer 34:0;O3	556.5293	[M+H]^+^	C_34_H_69_NO_4_	Cer 34:0;O3	SP
12	5.17	2.0857	**Cer 36:0;O3** ^#^	584.5599	[M+H]^+^	C_36_H_73_NO_4_	Cer 36:0;O3	SP
13	5.39	2.0522	**Cer 34:1;O2 B** ^#^	538.5181	[M+H]^+^	C_34_H_67_NO_3_	Cer 34:1;O2	SP
14	5.63	1.7453	PC 38:6 A ^#^	806.5691	[M+H]^+^	C_46_H_80_NO_8_P	PC 38:6	GP
15	5.73	1.7073	PC 36:4 ^#^	782.5695	[M+H]^+^	C_44_H_80_NO_8_P	PC 36:4	GP
16	5.97	1.6589	**PC 32:1** ^#^	732.5537	[M+H]^+^	C_40_H_78_NO_8_P	PC 32:1	GP
17	6.12	1.1106	PC 34:2 A4	780.5515	[M+Na]^+^	C_42_H_80_NO_8_P	PC 34:2	GP
		1.0793	PC 34:2 A3	796.5254	[M+K]^+^			
18	6.33	1.6936	**PC 33:1** ^#^	746.5687	[M+H]^+^	C_41_H_80_NO_8_P	PC 33:1	GP
19	6.37	1.4165	PC O-36:5	766.5744	[M+H]^+^	C_44_H_80_NO_7_P	PC O-36:5	GP
20	6.38	1.0167	PC O-36:4 A1	768.5908	[M+H]^+^	C_44_H_82_NO_7_P	PC O-36:4	GP
21	6.46	1.5990	PC O-38:6 ^#^	792.5894	[M+H]^+^	C_46_H_82_NO_7_P	PC O-38:6	GP
22	6.50	1.8693	PC O-34:3 ^#^	742.5741	[M+H]^+^	C_42_H_80_NO_7_P	PC O-34:3	GP
23	6.53	2.0849	PC O-36:4 ^#^	768.5900	[M+H]^+^	C_44_H_82_NO_7_P	PC O-36:4	GP
24	6.59	1.1784	PC 32:0	734.5693	[M+H]^+^	C_40_H_80_NO_8_P	PC 32:0	GP
25	6.60	1.5990	PC O-38:5 A ^#^	794.6057	[M+H]^+^	C_46_H_84_NO_7_P	PC O-38:5	GP
26	6.64	1.3519	PC 34:1	818.5933	[M+OAc]^−^	C_42_H_82_NO_8_P	PC 34:1	GP
27	6.64	2.2859	PC O-34:2 A ^#^	744.5895	[M+H]^+^	C_42_H_82_NO_7_P	PC O-34:2	GP
28	6.73	1.8068	PC O-36:3 ^#^	770.6046	[M+H]^+^	C_44_H_84_NO_7_P	PC O-36:3	GP
29	6.77	1.0375	PC 38:4 A	868.6082	[M+OAc]^−^	C_46_H_84_NO_8_P	PC 38:4	GP
		1.0957	PC 38:4 A1	810.6013	[M+H]^+^			
		1.0709	PC 38:4 A2	832.5826	[M+Na]^+^			
		1.0289	PC 38:4 A3	848.5560	[M+K]^+^			
30	6.83	1.0077	PC 36:2 A	844.6088	[M+OAc]^−^	C_44_H_84_NO_8_P	PC 36:2	GP
		1.1314	PC 36:2 A1	786.6031	[M+H]^+^			
		1.1153	PC 36:2 A2	808.5828	[M+Na]^+^			
		1.1557	PC 36:2 A3 ^#^	824.5563	[M+K]^+^			
31	6.87	1.1424	PS 41:4 ^#^	876.5696	[M+Na]^+^	C_47_H_84_NO_10_P	PS 41:4	GP
32	6.97	1.9089	**Cer 36:0;O2** ^#^	568.5651	[M+H]^+^	C_36_H_73_NO_3_	Cer 36:0;O2	SP
33	7.08	2.4984	PC O-40:6 ^#^	837.6194	[M+NH_4_]^+^	C_48_H_86_NO_7_P	PC O-40:6	GP
34	7.14	1.6514	PC O-38:5 B ^#^	794.6051	[M+H]^+^	C_46_H_84_NO_7_P	PC O:38:5	GP
35	7.31	1.6785	PC O-38:4 ^#^	796.6209	[M+H]^+^	C_46_H_86_NO_7_P	PC O-38:4	GP
36	7.47	1.3826	SM 42:3;O2 ^#^	811.6688	[M+H]^+^	C_47_H_91_N_2_O_6_P	SM 42:3;O2	SP
37	8.09	1.2309	SM 40:1;O2	787.6687	[M+H]^+^	C_45_H_91_N_2_O_6_P	SM 40:1;O2	SP
38	8.10	1.5929	**SM 42:2;O2** ^#^	813.6844	[M+H]^+^	C_47_H_93_N_2_O_6_P	SM 42:2;O2	SP
39	8.86	1.3595	SM 42:1;O2 ^#^	815.6997	[M+H]^+^	C_47_H_95_N_2_O_6_P	SM 42:1;O2	SP
40	9.75	1.4474	**DG 37:7** ^#^	647.4579	[M+Na]^+^	C_40_H_64_O_5_	DG 37:7	GL
41	9.84	1.0506	DG 36:0	647.5573	[M+Na]^+^	C_39_H_76_O_5_	DG 36:0	GL
42	11.06	1.1903	TG 50:4 A1	844.7381	[M+NH_4_]^+^	C_53_H_94_O_6_	TG 50:4	GL
		1.2262	TG 50:4 A2	849.6935	[M+Na]^+^			
		1.2083	TG 50:4 A3	865.6675	[M+K]^+^			
43	11.14	1.0506	TG 56:8 A2	941.6986	[M+K]^+^	C_59_H_98_O_6_	TG 56:8	GL
44	11.22	1.2595	TG 46:1	794.7225	[M+NH_4_]^+^	C_49_H_92_O_6_	TG 46:1	GL
45	11.25	1.4360	**TG 48:2 A1** ^#^	820.7384	[M+NH_4_]^+^	C_51_H_94_O_6_	TG 48:2	GL
		1.6212	**TG 48:2 A2** ^#^	825.6937	[M+Na]^+^			
		1.6244	**TG 48:2 A3** ^#^	841.6676	[M+K]^+^			
46	11.27	1.1443	TG 50:3 A1	846.7544	[M+NH_4_]^+^	C_53_H_96_O_6_	TG 50:3	GL
		1.3818	**TG 50:3 A2** ^#^	851.7097	[M+Na]^+^			
		1.4281	**TG 50:3 A3** ^#^	867.6836	[M+K]^+^			
		1.1385	TG 48:1 A2	827.7094	[M+Na]^+^	C_51_H_96_O_6_	TG 48:1	GL
		1.2088	TG 48:1 A3	843.6832	[M+K]^+^			
47	11.45	1.2724	TG 50:2 A2	853.7256	[M+Na]^+^	C_53_H_98_O_6_	TG 50:2	GL
		1.4536	**TG 50:2 A3** ^#^	869.6993	[M+K]^+^			
48	11.46	1.1703	TG 56:2	932.8628	[M+Na]^+^	C_59_H_110_O_6_	TG 56:2	GL
49	11.56	1.2680	TG 51:2 A2	867.7404	[M+Na]^+^	C_54_H_100_O_6_	TG 51:2	GL

* *m*/*z* mass-to-charge ratio; LPC: 1-acyl-sn-glycero-3-phosphocholines; GP: glycerophospholipids; FA: fatty acids; PA: 1,2-diacyl-sn-glycero-3-phosphates; Cer: *N*-acylsphinganines (dihydroceramides); SP: sphingolipids; PC: diacylglycerolphospho-cholines; PC O-: 1-alkyl,2-acylglycerophosphocholines; PS: diacylglycerolphospho-serines; SM: ceramide phosphocholines (sphingomyelins); DG: diacylglycerols; GL: glycerolipids; TG: triacylglycerols; N.D. not determined. VIP features contributing the most to the groups’ separation are in bold, while validated relevant features are denoted with a hash sign.

**Table 3:** Classification performance for the OPLS-DA models obtained for each partitioned subset of samples for female individuals.

Fold Number	*n* Comp	RMSECV	RMSEP	R_cv_^2^	R_p_^2^	VIP Selected	Calibration		Prediction	
							Accuracy	Misclass. Rate	Accuracy	Misclass. Rate
1	2	0.1635	0.0818	0.8933	0.9767	74	1	0.0	1	0.0
2	2	0.1619	0.1294	0.8956	0.9650	76	1	0.0	1	0.0
3	2	0.1608	0.1162	0.8969	0.9626	79	1	0.0	1	0.0
4	2	0.1613	0.1341	0.8970	0.9556	81	1	0.0	1	0.0
5	2	0.1207	0.2340	0.9432	0.9655	83	1	0.0	1	0.0

The cross-validation was performed for each training subset and model quality was measured by RMSECV, R_cv_^2^ (CV), as well as accuracy and misclassification rate for calibration. Prediction performance measure was given through RMSEP, R_p_^2^, and accuracy and misclassification rate for prediction. Number of selected variables was assigned according to VIP scores > 1 in all cases. n—number of components, RMSECV—root mean square error of cross-validation, RMSEP—root mean square error of prediction, R_cv_^2^—correlation coefficient of cross-validation, CV—cross-validation, R_p_^2^—correlation coefficient of prediction, VIP—variable importance in projection.

**Table 4:** Relevant *m*/*z* values according to the OPLS-DA model (combined positive and negative ion modes) assigned to the lipids, found differently in female schizophrenia patients (SCH-F) and healthy controls (C-F).

No.	Retention Time (min)	VIP Value	VIP Feature Assignment	Measured *m*/*z* *	Ion Mode Adduct	Proposed Formula	Lipid Assignment	Lipid Class
1	0.48	2.0627	*m*/*z* 293.1779 ^#^	293.1779	[M+OAc]^−^ or [M+H]^+^	C_15_H_22_O_2_ or C_17_H_26_O_4_	N.D.	Valerenic acid or Embelin
2	1.29	1.4333	LPC 18:2 A2 ^#^	542.3228	[M+Na]^+^	C_26_H_50_NO_7_P	LPC 18:2	GP
3	1.52	1.6482	**LPC 16:0 A** ^#^	554.3496	[M+OAc]^−^	C_24_H_50_NO_7_P	LPC 16:0	GP
		1.3009	LPC 16:0 A1 ^#^	496.3385	[M+H]^+^			
		1.2925	LPC 16:0 A2 ^#^	513.3667	[M+NH_4_]^+^			
		1.6891	**LPC 16:0 A3** ^#^	518.3202	[M+Na]^+^			
4	1.81	1.3138	LPC 18:1	522.3541	[M+H]^+^	C_26_H_52_NO_7_P	LPC 18:1	GP
5	2.15	1.0605	FA 24:0;O	402.3924	[M+NH_4_]^+^	C_24_H_28_O_3_	FA 24:0;O	FA
6	2.39	1.6640	FA 18:2 ^#^	279.2348	[M−H]^−^	C_18_H_32_O_2_	FA 18:2	FA
7	2.49	1.4026	LPC 18:0 A1 ^#^	524.3696	[M+H]^+^	C_26_H_54_NO_7_P	LPC 18:0	GP
		1.3593	LPC 18:0 A2 ^#^	582.3807	[M+OAc]^−^			
8	2.65	1.3760	LPC 18:0 B ^#^	524.3699	[M+H]^+^	C_26_H_54_NO_7_P	LPC 18:0	GP
9	2.89	1.2825	FA 20:3;O2 ^#^	337.2388	[M−H]^−^	C_20_H_34_O_4_	FA 20:3;O2	FA
10	3.21	1.4209	FA 18:1 ^#^	281.2506	[M−H]^−^	C_18_H_34_O_2_	FA 18:1	FA
11	4.52	1.2042	C30H58O3	484.4709	[M+H]^+^	C_30_H_58_O_3_	N.D.	N.D.
12	4.66	2.0896	**Cer 36:2;O3** ^#^	580.5288	[M+H]^+^	C_36_H_69_NO_4_	Cer 36:2;O3	SP
13	4.77	2.0538	**Cer 34:1;O2 A** ^#^	560.5026	[M+Na]^+^	C_34_H_67_NO_3_	Cer 34:1;O2	SP
14	4.84	2.0917	**Cer 34:2;O2** ^#^	536.5026	[M+H]^+^	C_34_H_65_NO_3_	Cer 34:2;O2	SP
15	5.17	1.5030	Cer 36:0;O3	584.5599	[M+H]^+^	C_36_H_73_NO_4_	Cer 36:0;O3	SP
16	5.21	1.1885	SM 34:2;O2 A2 ^#^	701.5587	[M+H]^+^	C_39_H_77_N_2_O_6_P	SM 34:2;O2	SP
17	5.39	1.8771	**Cer 34:1;O2 B** ^#^	538.5181	[M+H]^+^	C_34_H_67_NO_3_	Cer 34:1;O2	SP
18	5.61	1.5611	**PC 36:5** ^#^	780.5536	[M+H]^+^	C_44_H_78_NO_8_P	PC 34:2	GP
19	5.75	1.2836	SM 34:1;O2 A1 ^#^	703.5748	[M+H]^+^	C_39_H_79_N_2_O_6_P	SM 34:1;O2	SP
		1.2132	SM 34:1;O2 A2 ^#^	725.5564	[M+Na]^+^			
20	5.82	1.0849	PC 38:6 B	864.5768	[M+OAc]^−^	C_46_H_80_NO_8_P	PC 38:6	GP
21	5.92	1.0227	PC 30:0	706.5374	[M+H]^+^	C_38_H_76_NO_8_P	PC 30:0	GP
22	5.94	1.2440	SM 36:2;O2	729.5904	[M+H]^+^	C_41_H_81_N_2_O_6_P	SM 36:2;O2	SP
23	6.04	1.3172	PC 36:4 B5 ^#^	804.5515	[M+Na]^+^	C_44_H_80_NO_8_P	PC 36:4	GP
24	6.09	1.1320	PC 33:2 B ^#^	802.5619	[M+OAc]^−^	C_41_H_78_NO_8_P	PC 33:2	GP
25	6.12	1.2930	PC 34:2 A ^#^	816.5779	[M+OAc]^−^	C_42_H_80_NO_8_P	PC 34:2	GP
		1.1177	PC 34:2 A4 ^#^	780.5515	[M+Na]^+^			
		1.1252	PC 34:2 A3 ^#^	796.5254	[M+K]^+^			
26	6.33	1.2451	PC 33:1 ^#^	746.5687	[M+H]^+^	C_41_H_80_NO_8_P	PC 33:1	GP
27	6.37	1.4756	PC O-36:5 ^#^	766.5744	[M+H]^+^	C_44_H_80_NO_7_P	PC O-36:5	GP
28	6.46	1.3895	PC O-38:6 ^#^	792.5894	[M+H]^+^	C_46_H_82_NO_7_P	PC O-38:6	GP
29	6.50	1.6603	PC O-34:3 ^#^	742.5741	[M+H]^+^	C_42_H_80_NO_7_P	PC O-34:3	GP
30	6.52	1.2799	SM 36:1;O2	731.6060	[M+H]^+^	C_41_H_83_N_2_O_6_P	SM 36:1;O2	GP
31	6.53	1.6832	PC O-36:4 ^#^	768.5900	[M+H]^+^	C_44_H_82_NO_7_P	PC O-36:4	GP
32	6.60	1.5093	PC O-38:5 A ^#^	794.6057	[M+H]^+^	C_46_H_84_NO_7_P	PC O-38:5	GP
33	6.64	1.7027	PC O-34:2 A ^#^	744.5895	[M+H]^+^	C_42_H_82_NO_7_P	PC O-34:2	GP
34	6.71	1.2672	PC 38:4 A	868.6082	[M+OAc]^−^	C_46_H_84_NO_8_P	PC 38:4	GP
35	6.73	1.5719	PC O-36:3 ^#^	770.6046	[M+H]^+^	C_44_H_84_NO_7_P	PC O-36:3	GP
36	6.83	1.0584	PC 36:2 A	844.6088	[M+OAc]^−^	C_44_H_84_NO_8_P	PC 36:2	GP
37	6.83	1.0015	PC 35:2	830.5927	[M+OAc]^−^	C_43_H_82_NO_8_P	PC 35:2	GP
38	6.95	1.5164	PC O-32:1 ^#^	718.5736	[M+H]^+^	C_40_H_80_NO_7_P	PC O-32:1	GP
39	6.97	1.1207	Cer 36:0;O2 ^#^	568.5651	[M+H]^+^	C_36_H_73_NO_3_	Cer 36:0;O2	SP
40	7.08	1.5281	PC O-40:6 ^#^	837.6194	[M+NH_4_]^+^	C_48_H_86_NO_7_P	PC O-40:6	GP
41	7.11	1.5786	PC O-32:0 ^#^	720.5892	[M+H]^+^	C_40_H_82_NO_7_P	PC O-32:0	GP
42	7.14	1.5934	PC O-38:5 B ^#^	794.6051	[M+H]^+^	C_46_H_84_NO_7_P	PC O-38:5	GP
43	7.22	1.4271	PC O-34:1 ^#^	746.6050	[M+H]^+^	C_42_H_84_NO_7_P	PC O-34:1	GP
44	7.31	1.6867	PC O-38:4 ^#^	796.6209	[M+H]^+^	C_46_H_86_NO_7_P	PC O-38:4	GP
45	7.34	1.2701	SM 38:1;O2 ^#^	759.6370	[M+H]^+^	C_43_H_87_N_2_O_6_P	SM 38:1;O2	SP
46	7.46	1.4576	SM 40:2;O2 ^#^	785.6529	[M+H]^+^	C_45_H_89_N_2_O_6_P	SM 40:2;O2	SP
47	8.09	1.7065	SM 40:1;O2 ^#^	787.6687	[M+H]^+^	C_45_H_91_N_2_O_6_P	SM 40:1;O2	SP
48	8.49	1.2725	SM 41:1;O2 ^#^	801.6839	[M+H]^+^	C_46_H_93_N_2_O_6_P	SM 41:1;O2	SP
49	8.86	1.6592	SM 42:1;O2 ^#^	815.6997	[M+H]^+^	C_47_H_95_N_2_O_6_P	SM 42:1;O2	SP
50	9.75	1.1759	DG 37:7 ^#^	647.4579	[M+Na]^+^	C_40_H_64_O_5_	DG 37:7	GL
51	11.25	1.3020	**TG 48:2 A2** ^#^	825.6937	[M+Na]^+^	C_51_H_94_O_6_	TG 48:2	GL
		1.4037	**TG 48:2 A3** ^#^	841.6676	[M+K]^+^			
52	11.28	1.0147	TG 56:7 A1	922.7850	[M+NH_4_]^+^	C_59_H_100_O_6_	TG 56:7	GL
53	11.31	1.2363	TG 52:4 A1 ^#^	872.7703	[M+NH_4_]^+^	C_55_H_98_O_6_	TG 52:4	GL
		1.2912	TG 52:4 A2 ^#^	877.7257	[M+Na]^+^			
		1.2737	TG 52:4 A3 ^#^	893.6996	[M+K]^+^			
54	11.31	1.2322	TG 58:4 ^#^	956.8632	[M+NH_4_]^+^	C_61_H_110_O_6_	TG 58:4	GL
55	11.44	1.4314	**TG 48:1 A3** ^#^	843.6832	[M+K]^+^	C_51_H_96_O_6_	TG 48:1	GL
56	11.45	1.0780	TG 56:6 A1	924.8010	[M+NH_4_]^+^	C_59_H_102_O_6_	TG 56:6	GL
57	11.49	1.3233	TG 52:3 A1 ^#^	874.7863	[M+NH_4_]^+^	C_55_H_100_O_6_	TG 2:3	GL
		1.4266	TG 52:3 A2 ^#^	879.7414	[M+Na]^+^			
		1.2620	TG 52:3 A3 ^#^	895.7152	[M+K]^+^			
58	11.49	1.3423	TG 58:3 ^#^	958.8792	[M+NH_4_]^+^	C_61_H_112_O_6_	TG 58:3	GL
59	11.58	1.4998	CE 18:2 A1 ^#^	666.6174	[M+NH_4_]^+^	C_45_H_76_O_2_	CE 18:2	ST
		1.6483	CE 18:2 A2 ^#^	671.5727	[M+Na]^+^			
		1.7455	CE 18:2 A3 ^#^	1320.1560	[2M+Na]^+^			
60	11.62	1.0546	TG 48:0 A1	824.7697	[M+Na]^+^	C_54_H_100_O_6_	TG 48:02	GL

* *m/z* mass-to-charge ratio; N.D., not determined. LPC: 1-acyl-sn-glycero-3-phosphocholines; GP: glycerophospholipids; FA: fatty acyls; Cer: *N*-acylsphinganines (dihydroceramides); SP: sphingolipids; PC: diacylglycerolphosphocholines; SM: ceramide phosphocholines (sphingomyelins); PC O-: 1-alkyl,2-acylglycerophosphocholines; DG: diacylglycerols; GL: glycerolipids; TG: triacylglycerols; CE: cholesterol ester; ST: sterol lipids. VIP features contributing the most to the groups’ separation are in bold, while validated relevant features are denoted with hash sign.

## Data Availability

The lipidomics data presented in this study are unavailable due to privacy or ethical restrictions.

## Supplementary Materials

The following supporting information can be downloaded at: https://www.mdpi.com/article/10.3390/ijms251910266/s1.
