# Supplementary material for: LC–HRMS Lipidomic Fingerprints in Serbian Cohort of Schizophrenia Patients
Source: Int J Mol Sci. 2024 Sep 24;25(19):10266. doi: 10.3390/ijms251910266 (PMC11476971; doi:10.3390/ijms251910266)
Supplement: Supplementary file 1 [file ijms-25-10266-s001.zip › ijms-3170690-supplementary.pdf]

LC-HRMS Lipidomic Fingerprints in Serbian Cohort of Schizophrenia Patients

Suzana Marković[1,2], Milka Jadranin[3], Zoran Miladinović[4], Aleksandra Gavrilović[5], Nataša Avramović[6], Marija Takić[7], Ljubica Tasic[8], Vele Tešević[1] and Boris Mandić[1,*]

[1]University of Belgrade – Faculty of Chemistry, Studentski trg 12–16, 11000 Belgrade, Serbia; suzana.markovic@med.bg.ac.rs; vtesevic@chem.bg.ac.rs; borism@chem.bg.ac.rs
[2]University of Belgrade – Faculty of Medicine, Institute of Forensic Medicine, Deligradska 31a, 11000 Belgrade, Serbia; suzana.markovic@med.bg.ac.rs
[3]University of Belgrade – Institute of Chemistry, Technology and Metallurgy, Department of Chemistry, Njegoševa 12, 11000 Belgrade, Serbia; milka.jadranin@ihtm.bg.ac.rs
[4]Institute of General and Physical Chemistry, Studentski trg 12–16, 11158 Belgrade, Serbia; zmiladinovic@iofh.bg.ac.rs
[5]Special Hospital for Psychiatric Diseases "Kovin", Cara Lazara 253, 26220 Kovin, Serbia; gavrilovicaleksandra74@gmail.com
[6]University of Belgrade – Faculty of Medicine, Institute of Medical Chemistry, Višegradska 26, 11000 Belgrade, Serbia; natasa.avramovic@med.bg.ac.rs
[7]Institute for Medical Research, National Institute of Republic of Serbia, University of Belgrade, Center of Research Excellence for Nutrition and Metabolism, Group for Nutrition and Metabolism, Tadeuša Košćuška 1, 11000 Belgrade, Serbia; marija.takic@imi.bg.ac.rs
[8]Institute of Chemistry, Organic Chemistry Department, Universidade Estadual de Campinas, UNICAMP, Campinas 13083-970, SP, Brazil; ljubica@unicamp.br
* Correspondence: borism@chem.bg.ac.rs

Supplementary Material

Features ranking

Table S1. Relevant features ranked according to variable importance of mean decrease in accuracy obtained from Random Forest model aggregated by Borda_count method and *p* values of each feature obtained from F test comparative and independently from RF variable importance method.

No	Feature name	FStat	Lipid Class
1*	Cer 34:1;O2 A	3.18E-38	SP
2*	Cer 36:2;O3	1.32E-43	SP
3*	Cer 34:2;O2	4.01E-48	SP
4*	*m/z* 293.1779	2.31E-42	N.D.
5*,#	Cer 36:0;O3	3.48E-27	SP
6*,#	LPC 18:2 A2	1.26E-28	GP
7#	C30H58O3	1.2E-21	N.D.
8*,#	FA 20:3;O2	2.29E-23	FA
9*,#	LPC 16:0 A2	2.16E-25	GP
10*	FA 18:2	1.56E-24	FA
11#	LPC 18:2 A1	5.98E-16	GP
12#	FA 16:1	5.03E-19	FA
13#	LPC 18:2 A	8.03E-19	GP
14#	FA 14:0	6.39E-13	FA
15*,#	Cer 36:0;O2	4.43E-19	SP
16*	FA 18:1	3.87E-24	FA
17*	CE 18:2 A3	1.27E-25	ST
18*	Cer 34:1;O2 B	2.83E-18	SP
19	LPC 18:1	1.09E-10	GP
20*,#	LPC 16:0 A3	3.91E-11	GP
21#	FA 24:0;O	6.28E-18	FA
22*,#	DG 37:7	2.09E-17	GL
23	SM 36:2;O2	5.63E-16	SP
24*	CE 18:2 A2	3.6E-17	ST
25#	LPC 16:0 A	0.0000000203	GP
26	SM 38:2;O2	8.63E-14	SP
27*	TG 48:1 A3	3.1E-34	GL
28	DG 34:0 A2	0.0000103	GL
29	TG 56:7 A1	2.8E-12	GL
30*	SM 40:2;O2	1.56E-15	SP
31	LPC 20:4	0.0000000278	GP
32	Cer 34:0;O3	0.000368	SP
33*,#	SM 42:3;O2	1.34E-13	SP
34	DG 34:0 A1	0.0000953	GL
35	DG 36:0	0.000049	GL
36*	CE 18:2 A1	9.09E-13	ST
37	TG 56:6 A1	6.58E-12	GL
38*	LPC 18:0 B	0.00000411	GP
39*	TG 58:3	2.03E-16	GL

40*	LPC 16:0 A1	0.021	GP
41	TG 54:2 A3	1.83E-18	GL
42	TG 48:1 A2	2.45E-19	GL
43*	LPC 18:0 A1	0.0000143	GP
44*	TG 52:3 A2	1.46E-16	GL
45*	TG 58:4	3.06E-12	GL
46*	TG 52:4 A1	5.41E-13	GL
47*	TG 52:4 A2	2.54E-14	GL
48*,#	TG 48:2 A3	6.01E-22	GL
49	TG 56:7 A2	3.46E-15	GL
50*	LPC 18:0 A2	0.00000262	GP
51*	SM 38:1;O2	1.15E-11	SP
52	TG 56:6 A2	2.25E-13	GL
53*	TG 52:3 A1	3.97E-19	GL
54	TG 51:2 A2	5.14E-15	GL
55*	TG 52:4 A3	8.74E-15	GL
56	SM 36:1;O2	4.32E-11	SP
57	FA 20:4	0.0000000506	FA
58*,#	SM 34:2;O2 A2	2.63E-11	SP
59	TG 49:1	0.00813	GL
60*,#	TG 48:2 A2	3.04E-17	GL
61*	PC O-32:1	0.00000000526	GP
62	TG 52:5 A2	0.000151	GL
63	TG 50:2 A2	0.00058	GL
64	PC 36:4 B5	1.96E-10	GP
65*	SM 34:1;O2 A2	0.0000000251	SP
66*	PC O-34:1	0.00000000625	GP
67	TG 56:2	0.000065	GL
68	TG 56:5	5.17E-11	GL
69	PC 38:4 A	0.00000428	GP
70*	PC O-32:0	4.55E-10	GP
71*	SM 34:1;O2 A1	3.89E-12	SP
72	SM 41:2;O2	2.11E-11	SP
73*	SM 40:1;O2	1.78E-10	SP
74	TG 50:2 A1	0.00000138	GL
75*,#	SM 42:2;O2	2.63E-11	SP
76	TG 50:3 A1	0.00272	GL
77	TG 52:5 A1	0.000452	GL
78	LPS O-24:1;O	0.0000000566	GP
79	TG 52:5 A3	0.000225	GL
80	PA 25:0	0.000000099	GP
81	TG 48:1 A1	0.0342	GL
82*	TG 52:3 A3	4.02E-12	GL
83	FA 22:0	9.98E-11	FA
84	TG 48:0 A1	0.00000223	GL
85	TG 54:5 A1	0.000000248	GL

86	PC 36:4 B3	0.0000862	GP
87*[,#]	PC O-40:6	0.0000114	GP
88	PC 40:5 B1	0.00068	GP
89*	PC 36:4 B2	0.0000351	GP
90	TG 54:2 A2	0.0491	GL
91	TG 50:1 A3	0.00191	GL
92	PC 36:4 B1	0.0000000245	GP
93*	PC 34:2 A	0.000553	GP
94	PC O-34:2 B	0.0000337	GP
95[#]	TG 50:2 A3	0.994	GL
96	PC 38:4 A3	0.000141	GP
97	TG 51:2 A1	0.103	GL
98	PC 38:4 A1	0.00000477	GP
99	TG 56:8 A2	0.00495	GL
100	PC 34:1	0.00273	GP
101	TG 50:1 A1	0.0584	GL
102	SM 34:1;O2	0.0000000169	SP
103[#]	TG 50:3 A2	0.0324	GL
104	TG 52:1	0.0147	GL
105	PC 38:4 A2	0.000108	GP
106	TG 48:0 A2	0.0747	GL
107	MG 16:0	0.258	GL
108	PC 38:6 B	0.000000116	GP
109	PC 35:2	0.0256	GP
110*[,#]	PC O-34:3	0.0000731	GP
111	TG 54:5 A2	0.000000923	GL
112	TG 54:6 A2	0.000204	GL
113	TG 50:1 A2	0.597	GL
114	PC 32:0	0.0000413	GP
115	SM 33:1;O2	0.00000235	SP
116	PC O-36:4 A1	0.000011	GP
117	TG 58:2	0.0000493	GL
118	TG 54:5 A3	0.00000247	GL
119	TG 56:8 A1	0.00132	GL
120	TG 54:6 A1	0.000754	GL
121*	PC 33:2 B	0.000452	GP
122*[,#]	PC 33:1	0.209	GP
123*[,#]	PC O-38:6	0.00138	GP
124*[,#]	SM 42:1;O2	0.0000000392	SP
125*	PC 36:5	0.142	GP
126*	PC 34:2 A4	0.0000299	GP
127	PC 35:2 A1	0.277	GP
128	TG 51:2 A3	0.0133	GLGL
129	PC 34:0	0.78	GP
130	PC 36:4 B	0.0269	GP
131	PC 38:5	0.00683	GP

132	FA 21:4;O6	0.000222	FA
133[#]	TG 50:3 A3	0.0747	GL
134	SM 32:1;O2	0.000401	SP
135*	SM 41:1;O2	0.000000662	SP
136*	PC 34:2 A3	0.0000665	GP
137	PC 40:5 A1	0.0244	GP
138	PC 33:2 D	0.0124	GP
139	TG 50:4 A3	0.368	GL
140[#]	TG 48:2 A1	0.0000177	GL
141	PC 38:6 B1	0.0329	GP
142	LPI 20:3	0.161	GP
143	PC 36:3 A2	0.933	GP
144	TG 51:3	0.336	GL
145	TG 50:4 A2	0.25	GL
146	PC 40:7	0.00812	GP
147*[,#]	PC O-38:4	0.000251	GP
148*[,#]	PC O-38:5 B	0.000306	GP
149*[,#]	PC O-36:5	0.000961	GP
150	PI 40:3	0.391	GP
151	PC 40:6	0.000106	GP
152[#]	PS 41:4	0.41	GP
153[#]	C33H56O4	0.00168	N.D.
154[#]	PC 36:4	0.0949	GP
155[#]	PC 38:6 A	0.862	GP
156	Cer 42:1;O2	0.0214	SP
157	TG 50:4 A1	0.304	GL
158	PC 36:2 A2	0.129	GP
159	PC 34:1 A1	0.0013	GP
160	TG 54:2 A1	0.344	GL
161	PC 36:2 A	0.179	GP
162[#]	PC 36:2 A3	0.258	GP
163	PC 34:1 A2	0.00332	GP
164*[,#]	PC O-38:5 A	0.0103	GP
165	PC 40:4	0.0298	GP
166	PC 37:4	0.218	GP
167	PC 38:6	0.931	GP
168	PC 30:0	0.00154	GP
169	PC 35:4	0.598	GP
170	PC 33:2 C	0.00132	GP
171[#]	PC 32:1	0.127	GP
172*[,#]	PC O-36:4	0.0225	GP
173	TG 50:0	0.0000373	GL
174*[,#]	PC O-34:2 A	0.451	GP
175	PC 33:2 A	0.866	GP
176	PC 32:2	0.0125	GP
177*[,#]	PC O-36:3	0.791	GP

178	PC 38:2	0.746	GP
179	PS 39:3	0.00167	GP
180	TG 60:3	0.126	GL
181	DG 32:0	0.16	GP
182	TG 54:6 A3	0.0015	GL
183	PC 34:2 A1	0.0289	GP
184	PC 38:3	0.837	GP
185	TG 46:1	2.61E-14	GL
186	PC 34:3	0.997	GP
187	PC 36:3	0.499	GP
188	PC 36:2 A1	0.216	GP
189	TG 46:0	0.688	GL
190	PC 36:1	0.779	GP
191	PC 38:4	0.104	GP
192	PC 36:3 A1	0.473	GP

Cer: *N*-acylsphinganines (dihydroceramides); SP: sphingolipids; *m/z* – mass-to-charge ratio; LPC: 1-acyl-sn-glycero-3-phosphocholines; GP: glycerophospholipids; N.D.: not determined; FA: fatty acyds; CE: cholesterol ester; ST: sterol lipids; DG: diacylglycerols; GL: glycerolipids; SM: ceramide phosphocholines (sphingomyelins); TG: triacylglycerols; PC: 1,2-diacyl-sn-glycero-3-phosphocholine; LPS: 1-acyl-sn-glycero-3-phosphoserin; PA: 1,2-diacyl-sn-glycero-3-phosphates; * – validated features for female group; # – validated features for female group.

Variable importance of Random Forest classifier

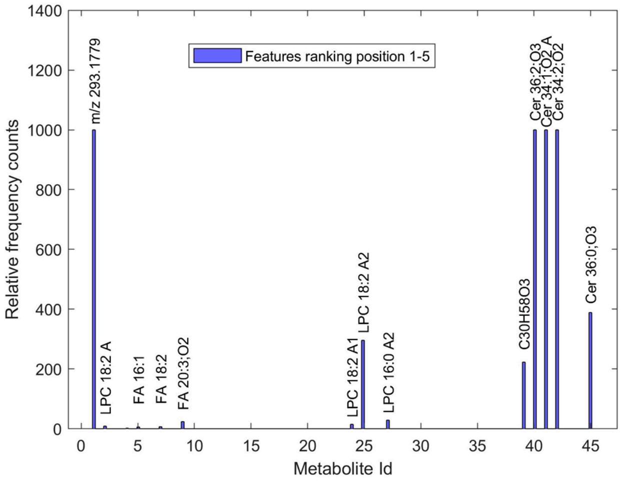

a)

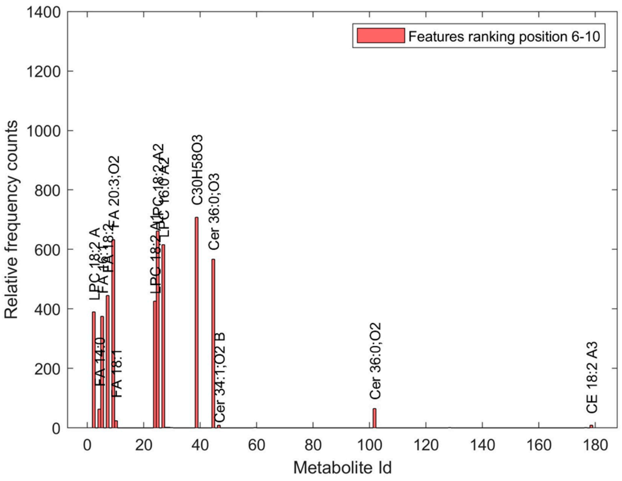

b)

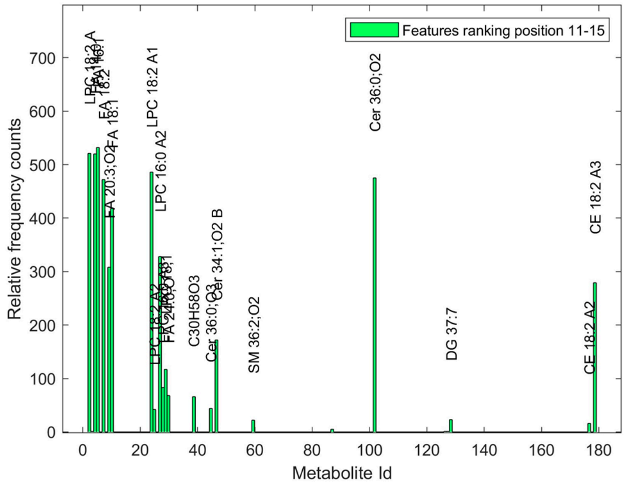

c)

Figure S1. a) Cumulative frequency distribution of variable indices from 1st to 5th ranking position in overall of 1000 generated lists; b) cumulative frequency distribution of variable indices from 6th to 10th ranking position in overall of 1000 generated lists; c) cumulative frequency distribution of variable indices from 11th to 15th ranking position in overall of 1000 generated lists. Each metabolite is assigned according to the initial feature position from the data table.

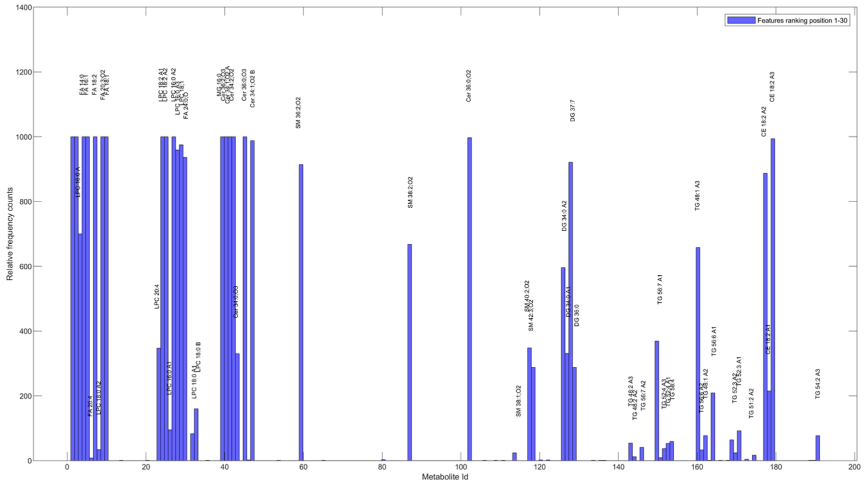

Figure S2. Cumulative frequency distribution of variable indices from 1st to 30th ranking position in overall of 1000 generated lists. Each metabolite is assigned according to the initial feature position from the data table.

Feature Validation

The complete procedure regarding proposed feature validation in this work has been performed in the following steps.

First, many VIP score lists have been created by independent randomly resampled observation of male and female subjects from cross-validated OPLS-DA models. The details about procedures of this proposed step could be adduced briefly as follows:

For both the male and female halves of the original data set, each of the ensuing sub-steps has been performed 10,000 times.

(1) All the data sets (male and female subjects) were repartitioned into 5-fold subgroups of samples and 5 independent calibration sets was assembled, by omitting exactly one-fold group of the samples (replicating in this way procedure for assembling of training blocks in section *2.3*).

(2) With the each of calibration sets, a regular OPLS-DA model was built using predetermined number of components from the section *2.3*, and the obtained VIP scores from each model with all variables were recorded in a $1 \times p$ vector, where p is the number of the variables.

(3) The lists of VIP scores were sorted in descending order, where the sorting indices of each variable’s position were stored in a separate ranking position vector that was the same size as the original VIP scores vector.

Repeating above sub-steps under the predefined number of iterations, resulting in matrix of dimension ($iter*fold \times p$) containing all ranking lists for each gender affiliation, where *iter* represents number of iteration and *fold* is number of folded groups. Aggregation of all obtained ranking lists, for both male and female subjects, was performed using borda count method [55]. As result one resulting ranking list for each gender is created necessary for proceeding in next step.

For features validation during developing of classification models different approaches are proposed [65]. One of them represents implementation of genetic algorithm (GA) in conjunction with PLS [66], method developed for validation of features ranking list based on the frequency of selection in population. In the final step of this procedure, a stepwise technique was performed, in which the variables are entered according to the “smoothed” value of the frequencies of selection, each time computing the RMSECV. The number of relevant variables was determined by visually inspecting the plot of the RMSECV versus the number of variables in the model, looking for the number of variables beyond which no “significant” decrease in RMSECV takes place [64]. This method was slightly adopted and utilized for the purpose of feature validation in this work. In our approach, rather than using GA to generate an ordered list of features, a resulting ranked list of features is based on the VIP scores.

The following sub-steps has been repeated 1000 times to validate the resulting features ranking lists that were obtained in the previous step:

(1) To ensure the most representative comparison between all models and discrimination in the number of features during modeling, an independent 5 folded CV partition of sample indices was conducted in each iteration.

(2) Maximum number of components for each gender subset of data were obtained based on the optimized models from the section 2.3.

(3) A set of independent cross-validate OPLS-DA models was constructed using all samples for a specific data set (male or female subjects). Each subset of features was obtained by cumulative addition, one feature at a time, based on increasing ordering position in the resulting ranking list, ranked from most relevant (at the top of the list) to least relevant (at the bottom).

(4) Each iteration’s developed cross-validated OPLS-DA models were used to determine the minimum RMSECV and optimal number of components for each feature subset in the resulting ranking lists. Furthermore, accuracy and misclassification error rates were also calculated from the confusion matrix for that specific CV OPLS-DA model, and they were recorded in the different data arrays with a size defined by the total number of iterations.

The final data structure incorporates arrays with the same size dimension ($1000 \times p$) for the minimum RMSECV, number of components, accuracy, and misclassification rate. The dimension of each ranked list is represented by p, which is also the number of features in the original data sets.

Averaged values of RMSECV, minimum number of components, accuracy and misclassification rate for each successive (cumulative) number of features were used as validation indicators for estimating position of relevant features in resulting ranked lists. Corresponding plots each of these diagnostics against the number of features in the CV model were presented in Figures S3 and S4.

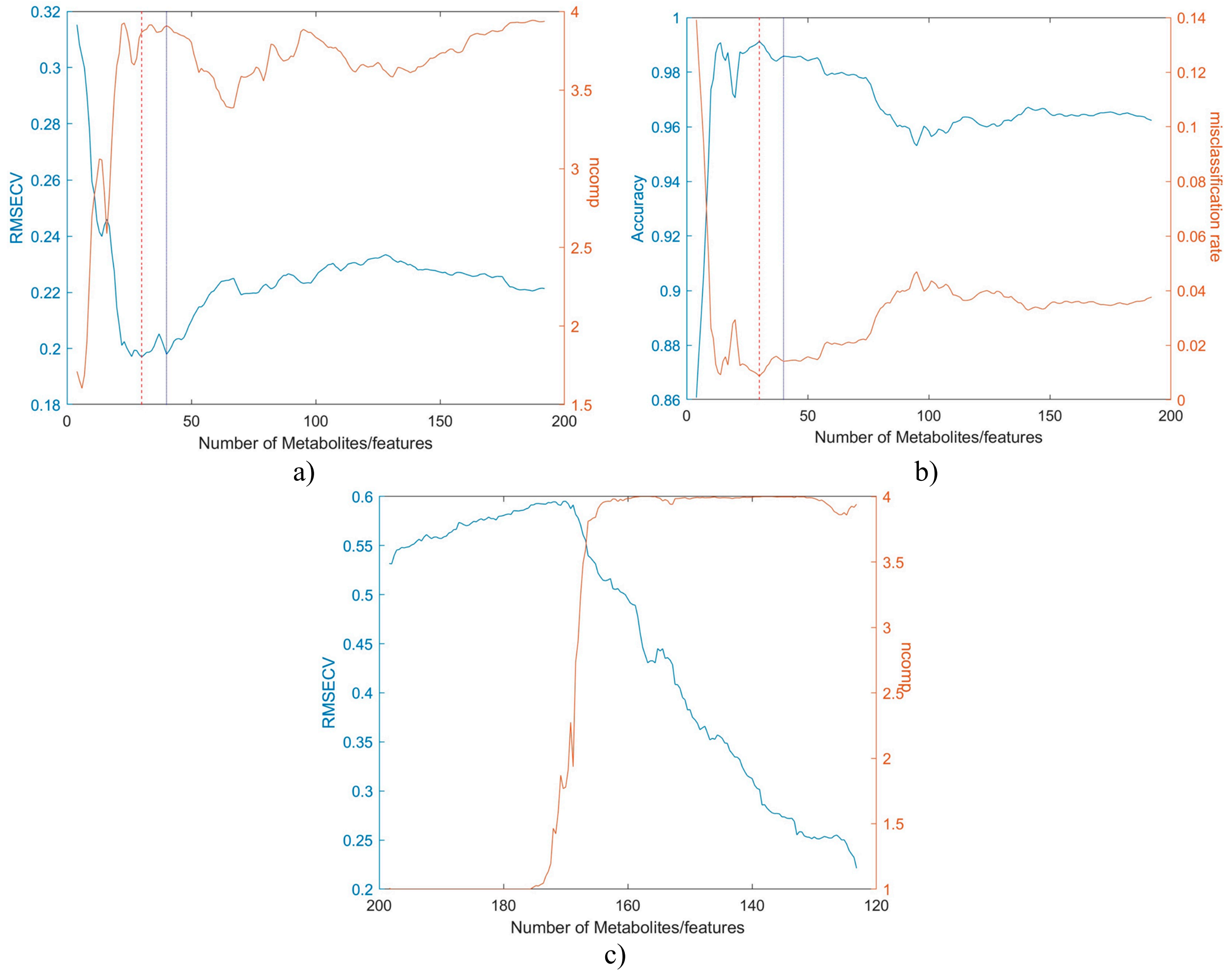

Figure S3. Several discrimination statistics performances are displayed in relation to the number of features in subsets that were created by adding features cumulatively, one by one, from the ranking list that was obtained for the male participants data sets. a) The average number of components is displayed mutually with the average values of the minimum RMSECV, where the number of metabolites within each subset are given in increasing order; b) Average values of accuracy presented in congruent with misclassification rate where number of metabolites in subsets are given in increasing order; c) Average values of minimum RMSECV presented jointly with mean number of components where the order of the number of metabolites in subsets are given in decreasing order. With dashed red line was assigned most extreme value for both accuracy and misclassification rate which was identified for first 30 ranked features; another value, which was primarily identified from minimum of RMSECV value, was assigned with doted blue line for the first 41 ranked features.

In Figure S3b were presented average values of accuracy and misclassification rate and in the Figure S3a were depicted average values for the minimum of RMSECV along with the number of components during successive addition of subset of ranked features in increasing order for the male subjects. The optimal range was shown by the minimum of the averaged values for RMSECV and misclassification rate, and at the same time, the maximum value for average accuracy, as indicated by dashed and dot vertical lines representing the least and maximum number from the resulting subsets of ranked features. These findings suggest that, for the data set containing only male individuals, subsets with ranking feature counts less than 30 and more than 41 might be producing more complex OPLS-DA models with greater “background correlation” [64]. As can be seen from Figure S3a, optimal number of LV for identified group

of relevant features was equal to 4, as was predetermined in the section 2.3. Likewise, similar findings are shown in Figures S4a and S4b, but for a data set that exclusively contains female participants. In this instance, the position of minimum RMSECV in Figure S4a indicates the presence of much more features that have been classified as more relevant features. According to these results, the best subset should contain the number of ranked features ranging between 55 and 61. Subsets of features out of this range could result in more complex OPLS-DA models. It should be noted that the results shown in Figure S4b, which compares the average values for accuracy and misclassification rate, indicate that all subsets with more than ten ranking features offer perfect classification in all cases (accuracy = 1, misclassification = 0). In that sense, results obtained from the Figure S4a provide much more valuable details regarding this matter, allowing in this way finding an optimal range of number of features from minimum of RMSECV. These findings also indicate RMSECV as a useful measure of performance parameter, even for classification models.

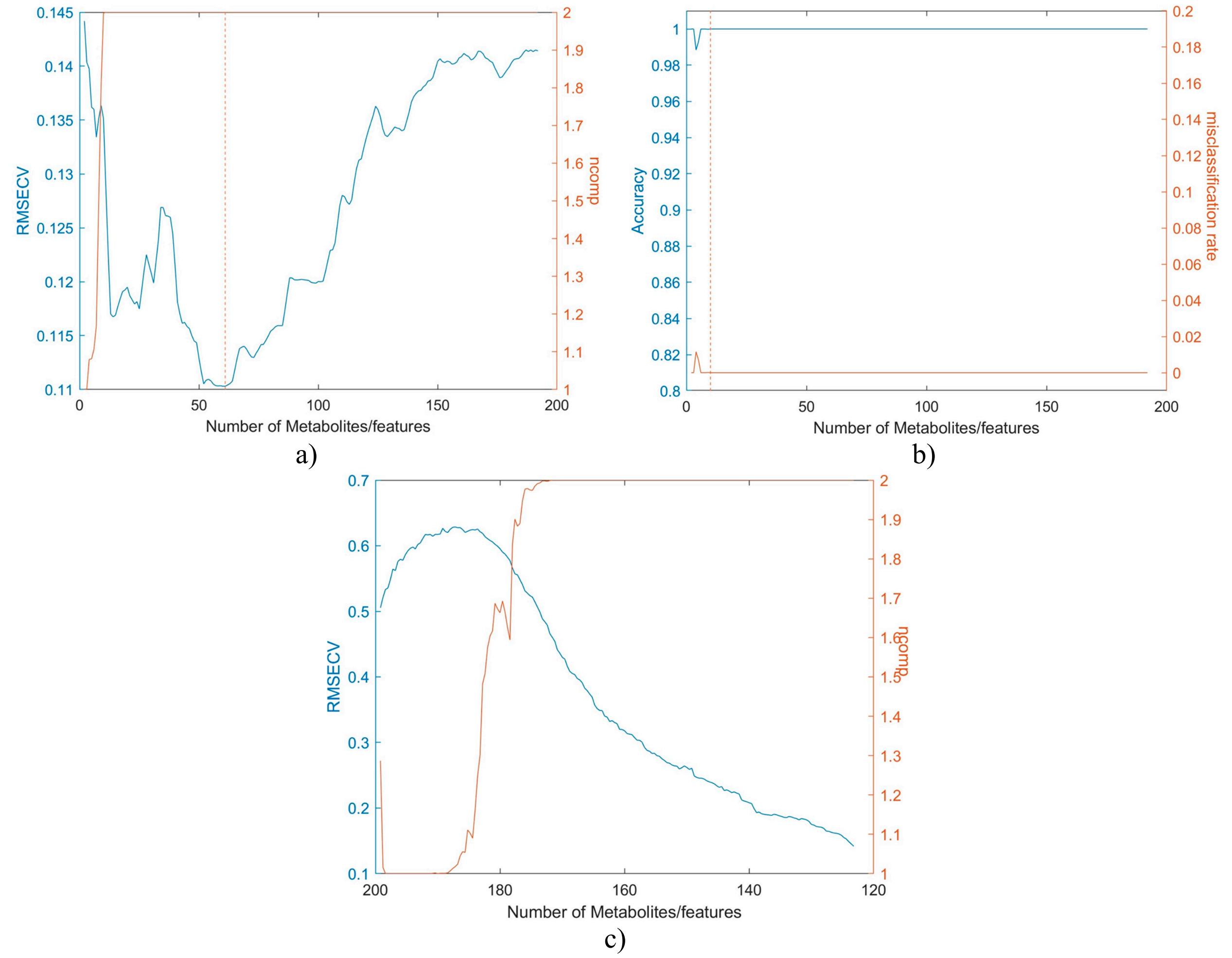

Figure S4. Several discrimination statistics performances are displayed in relation to the number of features in subsets that were created by adding features cumulatively, one by one, from the ranking list that was obtained for the female participants data sets. a) The average number of components is displayed mutually with the average values of the minimum RMSECV, where the number of metabolites within each subset are given in increasing order; b) Average values of accuracy presented in congruent with misclassification rate where the number of metabolites in subsets are given in increasing order; c) Average values of minimum RMSECV presented in parallel with mean number of

components where number of metabolites in subsets are given in decreasing order. With dashed red line was assigned the most extreme values for both accuracy and misclassification rate which was identified for the first 10 ranked features; in the case of the minimum of RMSECV value, with dashed red line the first 61 ranked features were assigned.

In Figures S3c and S4c were presented values for average minimum of RMSECV and number of components against the reverse ordered resulting ranked feature list for male and female subjects, respectively. In this representation, OPLS-DA models were composed by sub-setting features in reverse order of ranking position. Consequently, the RMSECV values were increased gradually for the subsets of the last 60 features (for the male subjects in Figure S3c, from 192 to 132), and the subset of the last 30 features (for the female subjects in Figure S4c, from 192 to 162). In both cases, the number of components related to the minimum RMSECV for these regions was equal to one. After that, the corresponding average number of LV progressively increases to its maximum, while the RMSECV (which in both cases showed its maximum) gradually decreases. This also indicates the correct ordering position for the subsequent cumulative addition of more significant features to the model. However, it should be noted that such presentation of reverse ordered ranking features, although demonstrate correct ordering of features in resulting ranking lists for both male and female subjects, also strongly indicate poor sensitivity of observed changes in average RMSECV during cumulative addition of more relevant sets of features.

Permutation test

Although obtained values of the diagnostic statistics, used in this work, show satisfying ranges, these values could be attained also purely by chance due to the favorable random choice of samples in corresponding training and/or validation data sets. Thus, each model has a possibility to possess in some degree so-called coincidental or chance correlation [64]. In addition, every regression data set contains structures which cause so-called “background correlation”. The latent structure in the predictor variable matrix X and the variable distribution in both X and the response variables are influential factors for the background correlation. An easy and efficient way to check how far a model is from being a coincidence correlation is by performing a permutation test [59], which at the same time allows to give a measure of the statistical significance of the diagnostic statistics (P-value).

Therefore, as a final confirmation of reliability and validity of OPLS-DA models assembled using all samples (for male and female part of data set) in first case and only selected features obtained during validation procedure in the second case, permutation test with 1000 iterations will be utilized. As in the previous scenario, independent 5-fold CV partition of samples in each of data sets was performed for each iteration. Labels of the response class variable are randomly permuted (shuffled), and the new classification model is calculated in each iteration. For each “permuted” OPLS-DA model, different diagnostic statistics were calculated (RMSECV, R^2Y, Q^2Y, Accuracy, Misclassification rate etc.) and recorded separately inside overall data structure. Besides, same diagnostic statistics were also recorded for the OPLS-DA model with “unpermuted” response class variable, to provide values for comparison with distribution of permuted values for each of the statistics in consideration.

The measure of correlation between each scrambled Y vector and the real-unscrambled one, was omitted from presented results. It could be expected for male and female data sets with given number of observations to be of some influence on the “background distribution”. However, since models composed with all features and selected number of features according to ascribed procedure, were compared in parallel, similar influence should be expected in each of these cases.

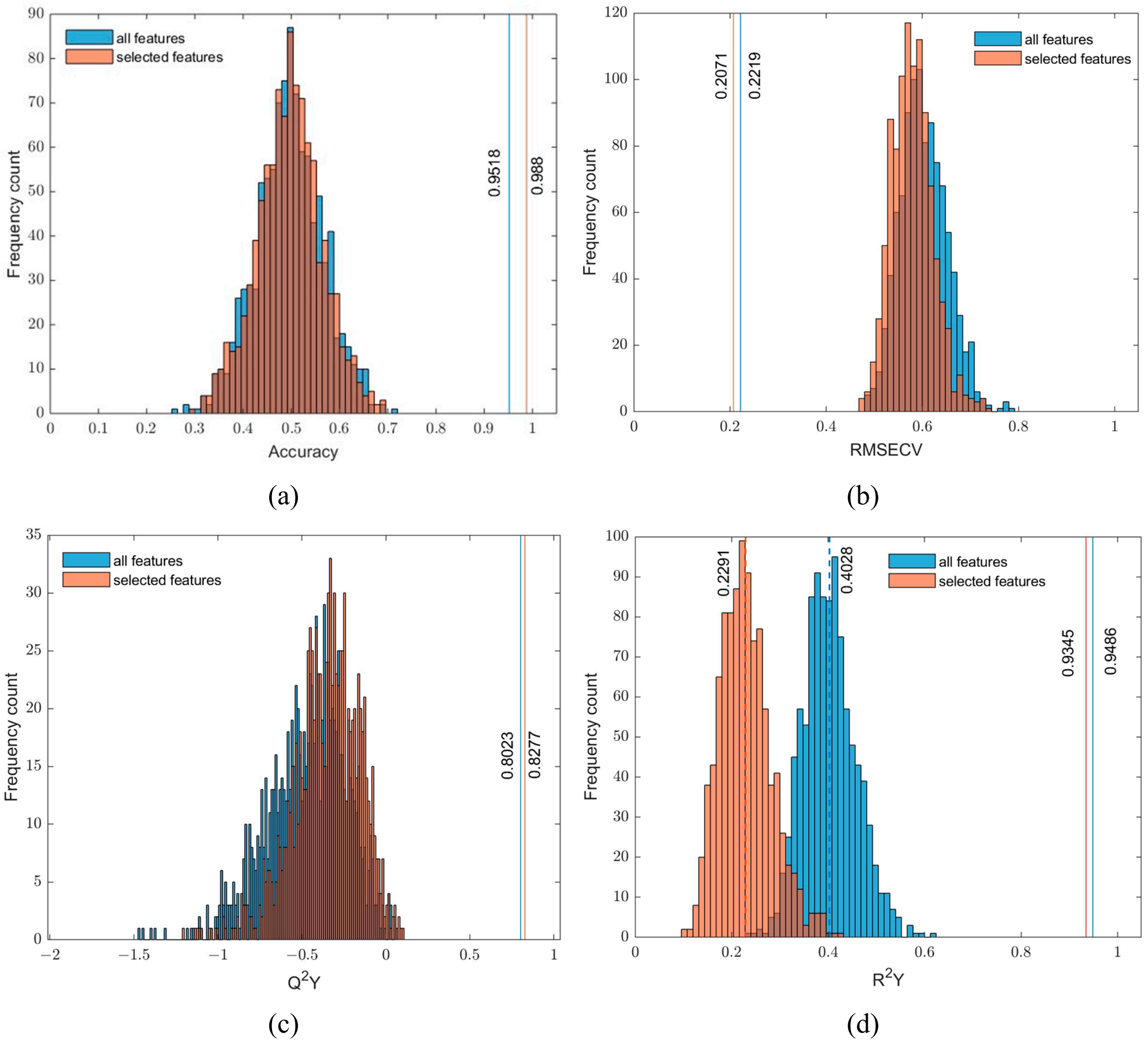

Figure S5. Permutation test for different diagnostic statistics for male part of samples in data set: a) Accuracy; b) RMSECV; c) Q^2Y; d) R^2Y. Null hypothesis H0 distribution was obtained using all features parallel with set of the first 41 selected features obtained from validation procedure. With solid lines along with corresponding numerical values corresponding unpermuted diagnostic statistics were indicated. Mean values of “background distribution” for R^2Y was additionally emphasized with dot-dashed vertical lines along with their numerical values.

Histograms in Figures S5 and S6 represent frequency of “background distribution” (null hypothesis H0 distribution) of corresponding measure for permuted Y variable 1000 times, while at the same time with corresponding sharp vertical lines were marked corresponding values for unpermuted real Y values models statistic measures. The background R^2Y and Q^2Y

as well as accuracy and RMSECV before and after variable selection (all features and only selected features) were recorded and used as the key measurement and presented simultaneously for male and female subjects' parts of data set. In all cases, except for R^2Y of male part of data set (Figure S5d), significant improvement in diagnostic statistics was observed, by comparison real unpermuted models with all included features and models with only selected number of features. The same was also true for the shift and shape of "background distribution" for permuted models, where the mean value of corresponding distribution was used as a measure of performance (assigned with corresponding dot-dashed vertical lines in Figures S5d and S6d). Moreover, by comparing the average R^2Y from permuted models based on the subsets of more significant variables with the average R^2Y of permuted models based on the full data set models, a clear drop in background correlation was observed (corresponding mean value for each of distribution was also assigned to the Figures S5d and S6d). Explanation for obtained results is in accordance with findings of Lindgren et al. [64], for distinct reduction in background R^2Y. Firstly, with a small number of variables *p,* the theoretical chance of having high background R^2Y is lower. Secondly, the variable subsets are optimized for explaining the real Y, which implies that their descriptive power for other Y-vectors is limited. Also, the lower dimensionality of the models with subset of features has a positive effect for the background R^2Y. No difference was found in comparing average Q^2Y, mainly because the average Q^2Y for the full data set models was already very low (see Figures S5c and S6c). In all cases, based on performed permutation tests, as can be seen from Figures S5 and S6, all considered diagnostic measures of performance were proved to be statistically significant ($p<0.001$).

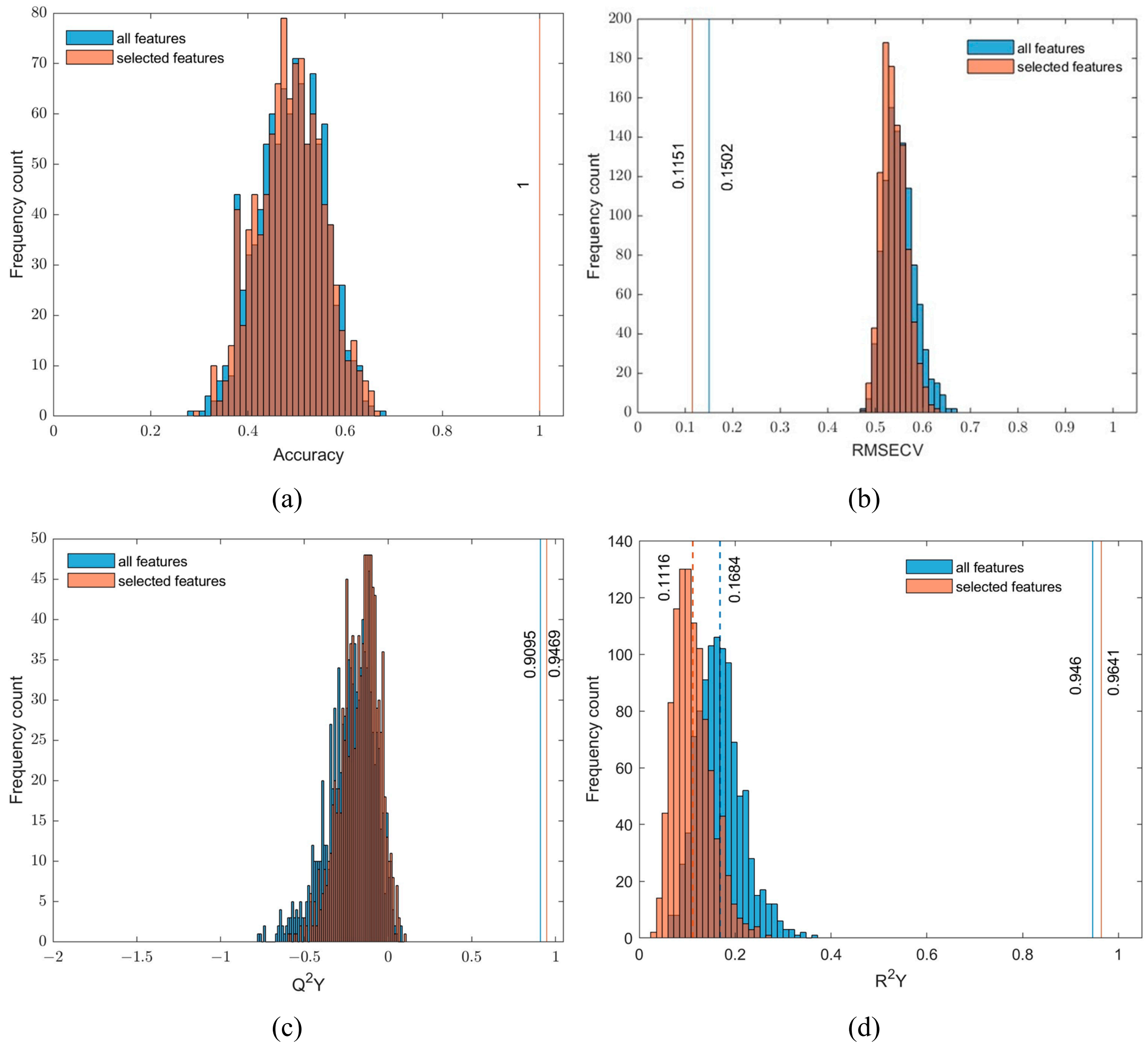

Figure S6. Permutation test for different diagnostic statistics for female part of samples in data set: a) Accuracy; b) RMSECV; c) Q^2Y; d) R^2Y. Null hypothesis H0 distribution was obtained using all features parallel with set of the first 61 selected features obtained from validation procedure. With solid lines along with corresponding numerical values corresponding unpermuted diagnostic statistics were indicated. Mean values of "background distribution" for R^2Y was additionally emphasized with dot-dashed vertical lines along with their numerical values.

Solvents and reagents

Chloroform (for HPLC, >99.8%, amylene stabilized, Sigma-Aldrich, France), methanol (LC-MS, Chromasolv™, ≥99.9%, Honeywell, Germany), 2-propanol (LiChrosolv®, hypergrade for LC-MS, Merck, Darmstadt, Germany), acetonitrile (LiChrosolv®, hypergrade for LC-MS, Merck, Darmstadt, Germany) and deionized water (18.2 MΩcm^{-1}, BarnsteadTM Smart2Pure™ Water Purification System, Thermo ScientificTM, USA) were used for lipids' extraction, dissolution and preparation of the mobile phases for the LC-HRMS analyses.

Ammonium formate (puriss. *p.a.*, eluent additive for LC-MS, Fluka, USA), formic acid (eluent additive for LC-MS, Fluka Analytical) and ammonium acetate (Optima® LC/MS, Fisher Chemical, USA) were used for the preparation of eluent additives for LC-HRMS.

Lipid extraction from blood serum samples

Non-polar metabolites were isolated from serum samples according to the methodology described by O'Brien et al. (2019) [84]. A total of 100 μL of serum sample thawed on ice were transferred to a 2.0 mL Eppendorf tube, and 100 μL of chloroform and 100 μL of methanol, both ice cold, were added. This mixture was mixed (vortex, MX-S) for 2 min, left to stand at −4 °C for 30 min, and then centrifuged for 15 min at 15,000 g (DLAB Centrifuge D2012 Plus) to yield upper (methanol/aqueous fraction) and lower phases (chloroform/lipid fraction). After centrifugation, 75 μL aliquots of the lower phase including lipids and chloroform were carefully sampled into 1.5 mL Eppendorf tubes, and solvent was evaporated till dryness in the mild nitrogen stream. The remaining components were resuspended in 1 mL of the mixture 2-propanol – acetonitrile – deionized water (2:1:1, *v*/*v*/*v*) at room temperature, transferred into 2 mL glass vials and subjected to the LC-HRMS analysis.

To detect possible sources of instrumental variation in the batch analysis of the samples, a third group of samples was analyzed, named quality control (QC), consisting of a pool of all samples to be analyzed, prepared following the same procedure using a pooled serum sample. Solvent blank samples (BS), prepared following the same procedure using deionized water, were injected at the beginning and at the end of each batch to allow detection and discarding of the background signals and impurities from the solvents or extraction protocol. A personal computer system running Agilent MassHunter software (revisions B.06.01 and B.07.00) was used for data acquisition and processing, respectively. All serum samples were prepared in triplicate.

Liquid chromatography-high resolution mass spectrometry (LC-HRMS) measurements

For untargeted lipidomics, prepared samples were injected into analysing system including liquid chromatograph (1290 Infinity LC system; Agilent Technologies, Waldbronn, Germany), with a quaternary pump, a column oven, and an autosampler, connected to the Quadrupole Time-of-Flight mass detector (6550 iFunnel Q-TOF MS, Agilent Technologies; Santa Clara, CA, USA) equipped with a dual spray Agilent Jet Stream (AJS) electrospray ion source. Separation of lipid compounds was performed using an Zorbax Eclipse Plus C18 column RRHD (100 mm × 2.1 mm; 1.8 μm, Agilent Technologies). Mobile phase was composed of a solvent A: water/ACN (40:60, v/v) and solvent B: IPA/ACN (90:10, v/v); both solvents were containing 10 mM ammonium formate and 0.1% formic acid (positive ionisation mode) or 10 mM ammonium acetate (negative ionisation mode). The following gradient program was used: 0–2 min 15–30% B, 2–2.5 min 30–48% B, 2.5–8.5 min 48–72% B, 8.5–11.5 min 72–99% B, 11.5–12 min 99 % B, 12.0–12.1 min 99–15% B, 12.1–15 min 15% B. The mobile phase flow rate was 0.60 $mLmin^{-1}$, the column temperature was 60 °C and the injection volume of samples and blanks was 2 μL (positive ionisation mode) or 4 μL (negative ionisation mode). After separation, the lipids were analysed using a mass detector. Positive and negative ion modes

were recorded (separately) and the instrument was operated in MS mode in the *m/z* range of 80 – 1,700, under following conditions: capillary voltage, 3,500 V, fragmentor voltage, 175 V, nozzle voltage, 1,000 V, skimmer 1, 65 V, octupole RF peak, 750 V, desolvation gas (nitrogen) temperature, 200 °C, desolvation gas (nitrogen) flow, 14 $Lmin^{-1}$, sheat gas (nitrogen) flow, 11 $Lmin^{-1}$. Ions *m/z* 121.05087300 and 922.00979800 in positive ion mode and 112.98558700, 966.00072500 and 1033.98810900 in negative ion mode were used as a lock mass for accurate mass measurements. Samples were recorded in consecutive batches for both positive and negative ionization modes, always after cleaning the ion source. Samples were randomly injected in the system to reduce the impact of small variations in instrument sensitivity during the measurements and to avoid any potential artificial sample clustering. In addition, QC samples were injected before the first and after every nine injections of lipid extract samples to monitor the analytical stability and performance of the system. Solvents blank samples were injected at the beginning and end of each batch to monitor background signals and contamination from the solvents or extraction protocol. A personal computer system running Agilent MassHunter software (revisions B.06.01 and B.07.00) was used for data acquisition and processing, respectively.

LC-HRMS data processing

The raw data (d) were converted to *mz*Data data format using Agilent MassHunter software (revision B.07.00) and then were processed using the XCMS online platform within the R statistical programming environment [86,89] for feature detection, retention time correction and alignment.

For the collected data, optimized XCMS parameters include centwave feature detection, orbiwarp retention time correction, minimum fraction of samples in one group to be a valid group = 0.50, P value thresholds for patients versus control samples = 0.05, isotopic ppm error = 15, width of overlapping *m/z* slices (mzwid) = 0.015, bandwidth grouping (bw) = 5, minimum peak width = 5 s, maximum peak width = 20 s. Thus, the raw data table of retention times, *m/z* values, and peak intensities were exported for further processing – cleaning of background noise, isotopic, and unrelated ions according to the data obtained by the Molecular Feature Extraction (MFE) tool in the MassHunter Qualitative Analysis Software (revision B.07.00, Agilent Technologies). For data extraction, the pre-set “small molecules (chromatographic)” algorithm was applied, with 200 counts as the limit for the background noise. In addition, to find co-eluting adducts of the same feature, the adduct settings (H^+, Na^+, K^+, neutral loss of water, and NH_4^+) for positive ionization were applied due to the ammonium formate in the mobile phase, and (H^-, CH_3COO^-) for negative ionization due to the ammonium acetate in the mobile phase. The option for “salt-dominated ion” was also applied. After removal of contaminants and background mass signals, dataset was filtered by selecting only features that were present in all samples within any group were considered.

The lipid molecules were assigned based on accurate mass measurements and databases – LIPID MAPS Structure Database (LMSD) [96] and Human Metabolome Database (HMDB) [97].

Data transformation and normalization

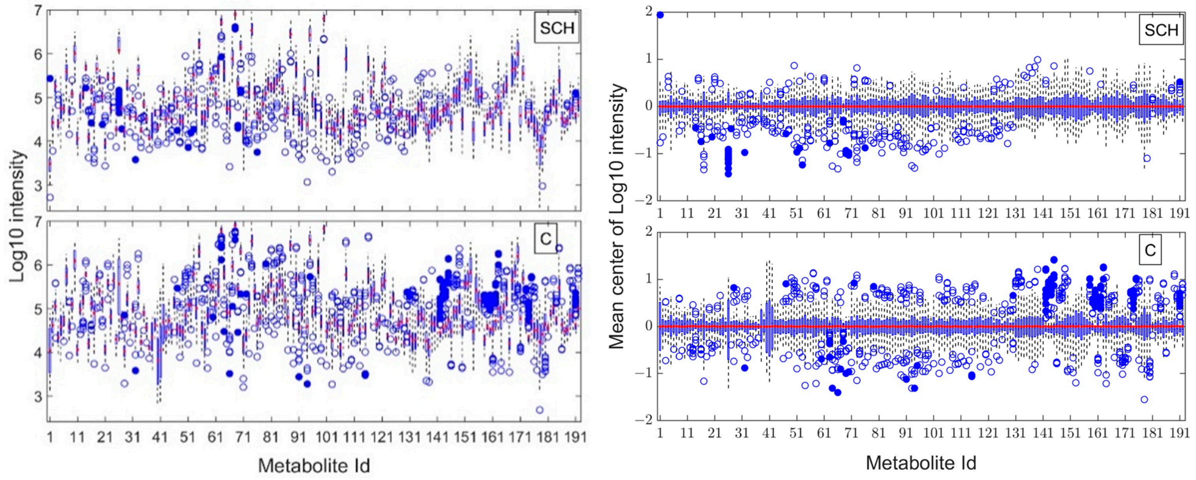

(a) (b)

Figure S7. a) Boxplot of log10-transformed data for SCH and C groups of individuals; b) Boxplot of log10-transformed and mean centered data for the same classes.

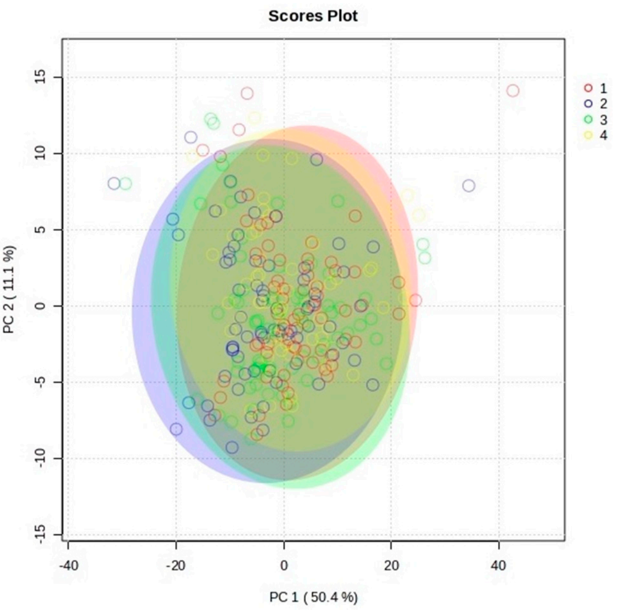

Figure S8. PCA model obtained for four consecutive batches.

Outlier detection

The diagnostic outlier map plot obtained from "robpca" Matlab routine [42–44] which is based on the score distances and orthogonal distances computed for each observation has been used to identify outlying observations. The ROBPCA distance plot for our data set using log10 transformation and autoscale centering and scaling of data is presented in Figure S9a. Number of relevant components for PCA model was predetermined based on the screeplot (which was also part of the LIBRA package). As relevant outliers, samples identified as "bad leverage points", which were positioned in the upper right corner, bordered by cut off lines for orthogonal and score distance of the outlier map, were assigned. Samples 145 and 13 lying near

these cut off lines could be regarded as outliers too. Moreover, sample 21 has significant orthogonal distance and therefore represents an orthogonal outlier, very similar to sample 13.

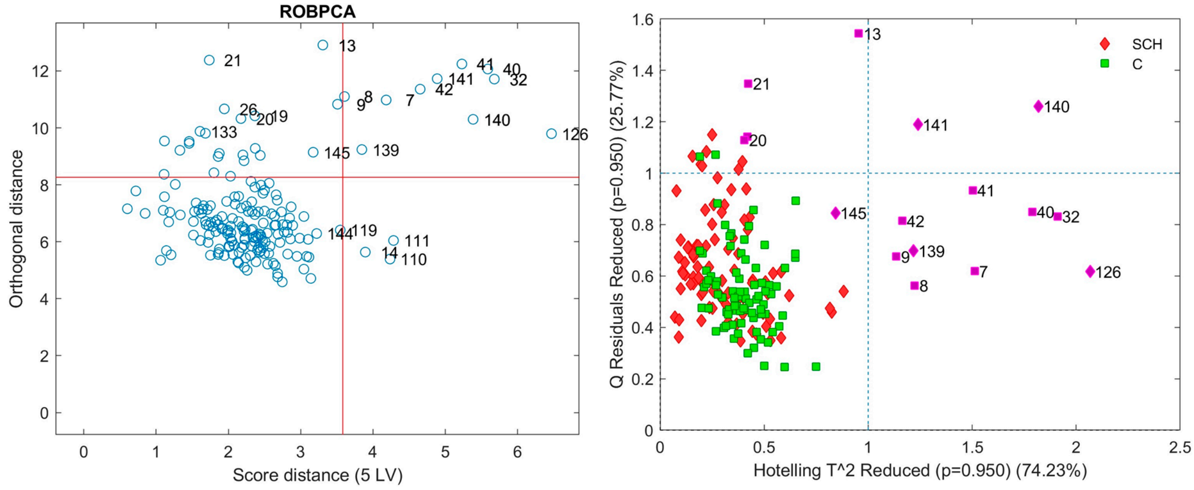

(a) (b)

Figure S9. a) ROBPCA outlier map of LC-HRMS data set composed using five PC components; b) IScatter plot, also called influence plot, of Q residuals vs. Hotelling's T^2 was presented for the same number of PC components. Both axes are represented as reduced Q and T^2 normalized statistics, divided by the confidence limit calculated from each model's particular data and parameters. Samples marked in pink color and assigned with number position in data set are identified as outliers, for easy comparison with ROBPCA results.

From definition of the score and orthogonal distance [42], these results could also be directly compared with the influence plot [94], obtained from classical PCA models, accomplished with the same number of PCA components as in the case of robust PCA. Results for such PCA model are presented in Figure S9b. Clearly, from this plot only samples 140 and 141 could be identified as potential outliers, confirming that in classical PCA analysis most outliers could be harder to detect due to the masking effect [95].

By comparison of contribution plot of selected samples (for both Q residuals, and Hotelling's T^2) with the rest of samples as a group of reference samples, none of the specific variables could be identified as responsible for contribution to the outlyingness. Overall, 14 distinctive outliers could be isolated from the original data set, although samples 21 and 145 could be treated as soft outliers.
